# Prevalence, Characterization, and Epidemiological Relationships between ESBL and Carbapenemase-Producing *Escherichia coli*, *Klebsiella pneumoniae,* and *Acinetobacter* spp. Isolated from Humans and the Kitchen Environment of Two Greek Hospitals

**DOI:** 10.3390/antibiotics13100934

**Published:** 2024-10-02

**Authors:** Anestis Tsitsos, Alexandros Damianos, Maria Boutel, Panagiota Gousia, Nikolaos Soultos, Anna Papa, Ilias Tirodimos, Vangelis Economou

**Affiliations:** 1 Laboratory of Animal Food Products Hygiene and Veterinary Public Health, School of Veterinary Medicine, Aristotle University of Thessaloniki, 54124 Thessaloniki, Greece; tsitanes@vet.auth.gr (A.T.); adamianb@vet.auth.gr (A.D.); soultos@vet.auth.gr (N.S.); 2 Hippokration General Hospital of Thessaloniki, 54642 Thessaloniki, Greece; mmpoutelag@gmail.com; 3 Department of Food Analytical and Research Laboratories of Thessaloniki, Hellenic Food Authority, 57001 Thermi, Greece; pgousia@efet.gr; 4 Laboratory of Microbiology, School of Medicine, Aristotle University of Thessaloniki, 54124 Thessaloniki, Greece; annap@auth.gr; 5 Laboratory of Hygiene, Social & Preventive Medicine and Medical Statistics, School of Medicine, Aristotle University of Thessaloniki, 54124 Thessaloniki, Greece; ityrodim@auth.gr

**Keywords:** antibiotic resistance, multidrug resistance, foodborne pathogens, ESKAPE, poultry, One Health

## Abstract

Background: Extended-spectrum-β-lactamase (ESBL) and carbapenemase-producing Enterobacterales and *Acinetobacter* spp. pose significant challenges as nosocomial pathogens, demonstrating resistance against various antimicrobials. Their presence in food suggests that hospital kitchens could serve as antibiotic resistance reservoirs leading to patients’ infection. Objectives: The aim of this study was to assess the prevalence and characteristics of β-lactam-resistant strains of *Escherichia coli*, *Klebsiella pneumoniae*, and *Acinetobacter* spp. isolated from the kitchen environment and from the staff of two Greek hospitals. Methods: Strains were recovered after selective isolation with β-lactams and were identified with MALDI–TOF MS. Antimicrobial susceptibility and presence of common β-lactamase genes were evaluated. Protein profiles were examined to analyze potential relationships of the strain with those from hospital patients. *E. coli* strains were further categorized into phylogenetic groups. Results: The overall prevalence in the kitchen environment was 4.5%, 1.5%, and 15.0% for *E. coli*, *K. pneumoniae,* and *Acinetobacter* spp., respectively, whereas the prevalence of *Acinetobacter* spp. in human skin was 4.0%. Almost all strains were multidrug-resistant. All *E. coli* strains were ESBL producers and belonged to phylogroups A and B1. All *K. pneumoniae* and seven *Acinetobacter* strains were carbapenemase-producers. A protein profile analysis showed relatedness between chicken and kitchen environment strains, as well as between kitchen environment and patient strains originated either from the same or from different hospitals. Conclusions: The results suggest that hospital kitchens may act as important pathogen hotspots contributing to the circulation of resistant strains in the hospital environment.

## 1. Introduction

Antimicrobial resistance (AMR) is a global public health challenge that tests the resilience of health systems worldwide. It significantly increases mortality and morbidity and drives up healthcare expenditures [1]. In 2019 alone, AMR was estimated to have contributed to 4.95 million deaths and 192 million Disability-Adjusted Life Years (DALYs) worldwide [2]. Malpractices, such as the overuse and misuse of antimicrobials and the prescription of broad-spectrum antibiotics contribute to the emergence of AMR bacteria [3]. A key factor in the development of AMR in healthcare facilities is the spread of multidrug-resistant (MDR) Gram-negative bacteria that produce β-lactamases, a resistance mechanism against β-lactam antibiotics encoded by genes often located on mobile genetic elements, capable of horizontal transfer among bacterial populations [4]. Predominant classes of β-lactamases include extended-spectrum β-lactamases (ESBLs), AmpC cephalosporinases, and carbapenemases. ESBL and AmpC-producing bacteria exhibit resistance to most β-lactam antibiotics, such as penicillins and cephalosporins. In contrast, carbapenemase-producing bacteria are also resistant to carbapenems, which are considered last-resort antibiotics, further limiting the treatment options [5,6]. Notable β-lactamase-producing Gram-negative bacteria include *Acinetobacter* spp. and species of the order Enterobacterales, such as *Escherichia coli* and *Klebsiella pneumoniae*.

*E. coli*, a commensal organism of the gastrointestinal tract, can be transformed into a significant opportunistic pathogen [7,8]. Improper use of antibiotics has led to the emergence of ESBL-producing Diarrheagenic *E. coli* (DEC) and Extraintestinal Pathogenic *E. coli* strains (ExPEC) that are often multidrug-resistant (MDR), limiting the available therapeutic options [9]. *K. pneumoniae* is one of the most common Gram-negative opportunistic pathogens, causing both hospital- and community-acquired infections worldwide [10]. The emergence of MDR, extremely drug-resistant (XDR), or even pan-drug-resistant (PDR) ESBL and carbapenemase-producing *K. pneumoniae* strains is a serious public health issue, significantly increasing the morbidity and mortality rates of hospital-acquired infections [11]. The production of carbapenemases confers resistance to almost all available β-lactam antibiotics, severely limiting treatment options [12]. *Acinetobacter* spp., particularly those in the *A. baumannii*–*calcoaceticus* complex (ACB complex), such as *A. baumannii*, *A. calcoaceticus*, *A. pittii*, *A. nosocomialis*, *A. seifertii*, and *A. dijkshoorniae*, are responsible for 2–10% of all Gram-negative hospital infections, with *A. baumannii* being the most prominent [10]. Both *A. baumannii* and *K. pneumoniae* are part of the ESKAPE group of pathogens (*Enterococcus faecium*, *Staphylococcus aureus*, *K. pneumoniae*, *A. baumannii*, *Pseudomonas aeruginosa*, *Enterobacter* spp.), which are leading causes of hospital-acquired infections and are known for their ability to acquire resistance to multiple antibiotic classes [13]. Infections caused by *A. baumannii* have become increasingly difficult to treat due to the emergence of carbapenem-resistant strains, which often harbor a diverse array of β-lactamase genes, notably OXA-type carbapenemase genes (e.g., OXA-51 like, OXA-23, OXA-58) [14].

Greece is endemic for ESBL and carbapenem-resistant *E. coli*, *K. pneumoniae*, and *A. baumannii*, with prevalence rates among the highest in Europe. According to 2021 data from antimicrobial resistance surveillance in Europe, the prevalence of third-generation cephalosporin-resistant and carbapenem-resistant *E. coli* in hospital patients in Greece was 21.7% and 1.1%, respectively. For *K. pneumoniae*, the prevalence rates were 80.4% and 73.7%, respectively. Similarly, the prevalence of carbapenem-resistant *A. baumannii* in Greece that year was 96.9% [15]. Despite numerous studies on the isolation of β-lactam-resistant *E. coli*, *K. pneumoniae*, and *A. baumannii* in hospital patients and their environments, there is limited information on the occurrence of ESBL, AmpC, and carbapenemase-producing bacteria in other hospital areas, such as hospital kitchens. β-lactam-resistant *E. coli*, *K. pneumoniae*, and *A. baumannii* have also been isolated from food products, particularly those of animal origin [16,17,18]. Thus, food may act as a vehicle for introducing resistant strains from the external environment into hospital kitchens. Moreover, resistant strains from hospital settings may enter the hospital kitchens via various pathways, such as equipment or personnel. Therefore, hospital kitchens may serve as AMR reservoirs that can transmit foodborne, resistant strains or AMR genes to the hospital environment and wards [19], especially through contaminated equipment or kitchen staff that comes in contact with patients, such as food service workers. This study aims to evaluate the prevalence and seasonal fluctuations of β-lactamase-producing *E. coli*, *K. pneumoniae*, and *Acinetobacter* spp. of the ACB complex in the kitchens and their staff in two Greek hospitals following the One Health principle.

## 2. Results

### 2.1. Prevalence and Seasonal and Regional Variations of Strains

**Figure 1:**
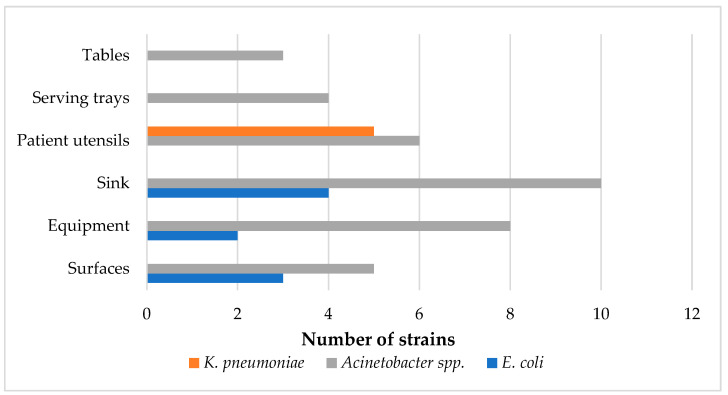
Number of strains isolated across the sampling points in the hospital environment.

Ceftazidime- and meropenem-resistant *E. coli*, *K. pneumoniae*, or *Acinetobacter* spp. were isolated from 42 of the 200 (21.0%) samples from the kitchen environment. Only one bacterial genus was isolated from each sample. Regarding *E. coli*, nine samples contained resistant *E. coli* strains (4.5%, 95% CI = 2.1%–8.4%). More specifically, nine ceftazidime-resistant strains were isolated from sinks (n = 4, 10.0%), surfaces (n = 3, 7.5%), and equipment (n = 2, 5%) (Figure 1). Most *E. coli* strains were recovered from chicken-related samples, except from one from a vegetable cutting board. Concerning *K. pneumoniae*, five meropenem-resistant strains were isolated from three samples (1.5%, 95% CI = 0.3%–4.3%), all from utensils used by hospital patients (7.5%) (Figure 1). In addition, 30 samples carried resistant *Acinetobacter* spp. (15.0%, 95% CI = 10.4%–20.7%). Resistant *Acinetobacter* spp. was found in all different types of kitchen samples, including surfaces (n = 5, 12.5%), equipment (n = 7, 17.5%), sinks (n = 8, 20.0%), utensils (n = 4, 10.0%), serving trays (n = 3, 12.5%), and tables (n = 3, 18.8%) (Figure 1). Positive samples were mostly associated with cheese, vegetables, red meat, food preparation, and washing up, with one positive sink used by kitchen staff for personal use. Among the thirty-six *Acinetobacter* spp. strains, twenty-four were typed as *A. baumannii*, six as *A. pittii*, four as *A. calcoaceticus*, and two as *A. nosocomialis*. Of the seven meropenem-resistant *Acinetobacter* spp., five were *A. baumannii*, one was *A. calcoaceticus*, and one was *A. pittii*, isolated from three utensils used by hospital patients, two sinks (cheese, vegetables), one serving tray, and one vegetable knife.

Additionally, 14 ceftazidime-resistant *E. coli* strains were isolated from 14 chicken samples, out of a total of 40 samples collected (35.0%, 95% CI = 20.6%–51.7%), with no *K. pneumoniae* or *Acinetobacter* spp. isolated from chicken samples (95% CI = 0.0%–8.8%). Regarding the human samples, one *A. baumannii* and one *A. pittii* strain were isolated from the skin of one chef (4.0%, 95% CI = 0.1%–20.4%). No *Acinetobacter* spp. was found in the oropharynx of hospital staff (95% CI = 0.0%–13.7%), and no *E. coli* or *K. pneumoniae* strains were found on the skin or in the oropharynx of hospital staff (95% CI = 0.0%–13.7%) (Tables S1–S3).

Regarding seasonal variation, no significant differences were observed concerning the prevalence of resistant *K. pneumoniae* and *Acinetobacter* spp. in the hospital kitchen environment across seasons (p = 0.06 and 0.1, respectively). *Acinetobacter* spp. strains were isolated as follows: six in spring (10.0%, 95% CI = 3.3%–21.8%), seventeen in summer (24.0%, 95% CI = 13.1%–38.2%), nine in autumn (18.0%, 95% CI = 8.6%–31.4%), and four in winter (8.0%, 95% CI = 2.2%–19.2%). For *K. pneumoniae*, five strains were found in spring (6.0%, 95% CI = 1.3%–16.5%), with no strains in other seasons (95% CI = 0.0%–7.1%). However, seasonality significantly affected the prevalence of *E. coli* in the hospital kitchen environment (*p* = 0.03 < 0.05). Most *E. coli* strains were isolated in the summer (six strains, 12.0%, 95% CI = 4.5%–24.3%), followed by spring (two strains, 4.0%, 95% CI = 0.5%–13.7%) and winter (one strain, 2.0%, 95% CI = 0.1%–10.6%), with none in autumn (95% CI = 0.0%–7.1%). However, no significant differences were observed in the prevalence of *E. coli* in chicken samples (*p* = 0.08) and *Acinetobacter* spp. in human samples (*p* = 1) across seasons. *E. coli* strains from chicken samples were isolated as follows: spring (five strains, 50%, 95% CI = 18.7%–81.3%), summer (six strains, 60.0%, 95% CI = 26.2%–87.8%), autumn (two strains, 20%, 95% CI = 2.5%–55.6%), and winter (one strain, 10%, 95% CI = 0.3%–44.5%). All *Acinetobacter* spp. strains from the skin of one chef were isolated in summer (Tables S1–S3).

Differences in prevalence were also noted between the two hospitals. The prevalence of *Acinetobacter* spp. in the kitchen environment was significantly higher in the Central Macedonia Region hospital (23 strains, 21.0%, 95% CI = 13.5%–30.3%) compared to the Epirus Region hospital (13 strains, 9.0%, 95% CI = 4.2%–16.4%) (*p* = 0.03 < 0.05). No significant differences were observed in the prevalence of resistant *E. coli* and *K. pneumoniae* in the hospital kitchen environment between the two Regions (*p* = 0.5 and 1.00, respectively). Six (6.0%, 95% CI = 2.2%–12.6%) and three (3.0%, 95% CI = 0.6%–8.5%) strains of *E. coli* were isolated from the Central Macedonia and Epirus Regions, respectively; four *K. pneumoniae* strains were isolated from the Epirus Region (2.0%, 95% CI = 0.2%–7.0%) and one *K. pneumoniae* strain was isolated from the Central Macedonia Region (1.0%, 95% CI = 0.0%–5.4%). Regarding chicken samples, the prevalence of *E. coli* was significantly higher in the Central Macedonia Region compared to the Epirus Region (*p* = 0.02 < 0.05). Specifically, 11 *E. coli* strains were recovered from Central Macedonia (55.0%, 95% CI = 31.5%–76.9%), whereas 3 *E. coli* strains were isolated in Epirus (15.0%, 95% CI = 3.2%–37.9%). In human samples, *Acinetobacter* strains were recovered from the skin of one chef in the Central Macedonia hospital. No significant differences were observed in the prevalence of *Acinetobacter* spp. in hospital staff between the two Regions (*p* = 1) (Tables S1–S3).

### 2.2. Antimicrobial Susceptibility Testing of the Isolated Strains

**Figure 2:**
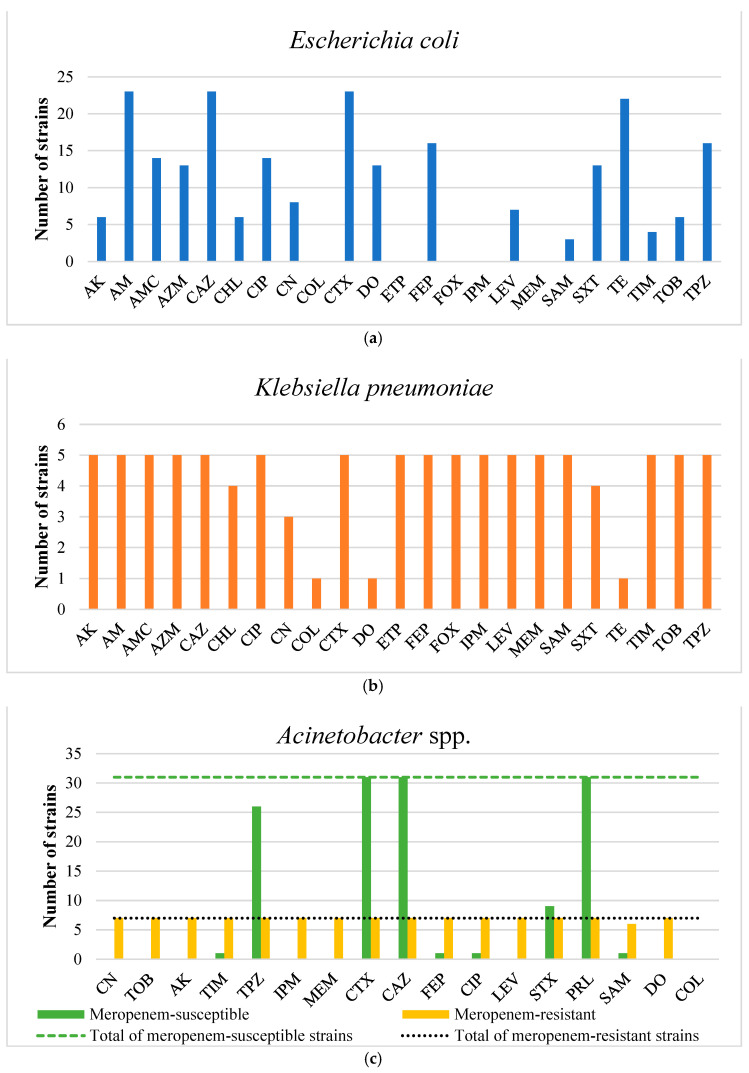
Number of AMR strains of *E. coli* (n = 23) (**a**), *K. pneumoniae* (n = 5) (**b**), and *Acinetobacter* spp. (n = 31 MEM-susceptible and 7 MEM-resistant) (**c**).

Regarding the *E. coli* strains, the spectrum of antibiotic resistance varied from 0.0% [against meropenem (MEM), ertapenem (ETP), imipenem (IPM), cefoxitin (FOX), and colistin (COL)] to 100.0% [against ceftazidime (CAZ), cefotaxime (CTX), and ampicillin (AM)]. High resistance rates were also noted for tetracycline (TE, 95.7%, n = 22), cefepime (FEP, 69.6%, n = 16), and piperacillin–tazobactam (TPZ, 69.6%, n = 16). The strains displayed resistance to varying numbers of antibiotics, ranging from 4 (8.7%, n = 2) to 18 antibiotics (4.4%, n = 1). Consequently, all strains (100.0%) were classified as MDR, showing resistance to at least three different antibiotic categories. A total of 21 distinct AMR profiles were identified (Figure 2, Table S1).

For the non-clinical *K. pneumoniae* strains, antibiotic resistance percentages were 20.0% [against TE, doxycycline (DO), and COL], 60.0% [against gentamicin (CN)], 80.0% [against trimethoprim–sulfamethoxazole (SXT) and chloramphenicol (CHL)], and 100.0% [against amikacin (AK), AM, amoxicillin–clavulanic acid (AMC), azithromycin (AZM), CAZ, CTX, ciprofloxacin (CIP), levofloxacin (LEV), ETP, IPM, MEM, FEP, FOX, ampicillin–sulbactam (SAM), ticarcillin–clavulanic acid (TIM), tobramycin (TOB), and TPZ]. All strains were resistant to carbapenems, and one strain was additionally resistant to colistin. The MIC values for colistin ranged from 0.125 to 8 μg/mL, with a median of 0.25 μg/mL (IQR = 0.75). All strains (100.0%) were classified as MDR. Four distinct antimicrobial resistance profiles were identified (Figure 2, Table S2).

Concerning the non-clinical *Acinetobacter* strains, all seven meropenem-resistant *Acinetobacter* spp. were characterized as XDR, exhibiting resistance to all antimicrobials tested except colistin (all strains) and ampicillin–sulbactam (one strain). Two antimicrobial resistance profiles were identified, [AK, CAZ, CIP, CN, CTX, DO, FEP, IPM, LEV, MEM, PRL, SAM, SXT, TIM, TOB, TPZ] and [AK, CAZ, CIP, CN, CTX, DO, FEP, IPM, LEV, MEM, PRL, SXT, TIM, TOB, TPZ], observed in six and one strain, respectively. Among the meropenem-susceptible *Acinetobacter* spp., all strains were resistant to CAZ, CTX, and PRL (100.0%), with most also showing high resistance rates to TPZ (83.9%, n = 26). Conversely, all meropenem-susceptible strains demonstrated sensitivity to COL, IPM, MEM, CN, TOB, AK, LEV, and DO. The median MIC for colistin was 0.125 μg/mL (IQR = 0.0625) for meropenem-resistant strains and 0.25 μg/mL (IQR = 0.125) for meropenem-susceptible strains. The meropenem-susceptible strains displayed varying degrees of resistance, with the lowest showing resistance to 3 antibiotics (16.1%, n = 5) and the highest to 7 antibiotics (3.2%, n = 1). Consequently, most meropenem-susceptible strains were classified as MDR (80.7%, n = 25). A total of six distinct antimicrobial resistance profiles were identified for the meropenem-susceptible strains. The most prevalent profile was [CAZ, CTX, PRL, TPZ], shared by nearly half of the strains (48.4%, n = 15) (Figure 2, Table S3).

Concerning the clinical strains, the *K. pneumoniae* strains exhibited extensive resistance to most antibiotics tested. Specifically, all strains showed resistance to CAZ, CTX, AM, AMC, FEP, FOX, SAM, TIM, TPZ, MEM, ETP, CIP, LEV, SXT, and AZM. High resistance levels were observed for AK and IPM (89.5%); TOB, TE, and DO (84.2%); and CN (79.0%). Conversely, the lowest resistance levels were seen for CHL (31.58%, n = 6) and COL (10.5%, n = 2). The MIC values for colistin ranged from 0.06 to ≥32 μg/mL, with a median of 0.125 μg/mL (IQR = 0.44). All strains (100.0%) were classified as MDR. In total, nine distinct antimicrobial resistance profiles were identified. The most commonly shared resistance profiles were (a) [AK, AM, AMC, AZM, CAZ, CIP, CN, CTX, DO, ETP, FEP, FOX, IPM, LEV, MEM, SAM, SXT, TE, TIM, TOB, TPZ] (shared by six strains), (b) [AK, AM, AMC, AZM, C, CAZ, CIP, CN, CTX, DO, ETP, FEP, FOX, IPM, LEV, MEM, SAM, SXT, TE, TIM, TOB, TPZ] (shared by four strains), (c) [AK, AM, AMC, AZM, CAZ, CIP, CN, CTX, DO, ETP, FEP, FOX, LEV, MEM, SAM, SXT, TE, TIM, TOB, TPZ] (shared by two strains), and (d) [AM, AMC, AZM, C, CAZ, CIP, CTX, DO, ETP, FEP, FOX, IPM, LEV, MEM, SAM, SXT, TE, TIM, TPZ] (shared by two strains). No common resistance profiles were observed between the clinical and non-clinical *K. pneumoniae* strains. Regarding the clinical *A. baumannii* strains, seven strains (43.8%) were categorized as PDR, exhibiting resistance to all antimicrobials tested. The remaining nine strains (56.3%) were classified as XDR, displaying resistance to all antimicrobials except for colistin. Two distinct antimicrobial resistance profiles were identified: [AK, CAZ, CIP, CN, COL, CTX, DO, FEP, IPM, LEV, MEM, PRL, SAM, SXT, TIM, TOB, TPZ] and [AK, CAZ, CIP, CN, CTX, DO, FEP, IPM, LEV, MEM, PRL, SAM, SXT, TIM, TOB, TPZ], shared by seven and nine strains each. The MIC values for colistin ranged from 0.125 to 16 μg/mL, with a median of 1.5 μg/mL (IQR = 4.875). The clinical strains had different resistance profiles compared to the non-clinical meropenem-susceptible strains, showing resistance to a significantly higher number of antimicrobials. However, six out of seven (85.7%) non-clinical meropenem-resistant strains shared the same resistance profile as the nine clinical strains (56.3%) that were susceptible only to colistin. The shared resistance profile was [AK, CAZ, CIP, CN, CTX, DO, FEP, IPM, LEV, MEM, PRL, SAM, SXT, TIM, TOB, TPZ] (Tables S2 and S3).

### 2.3. Phenotype of β-Lactam Resistance and Molecular Screening of β-Lactamase Genes

**Figure 3:**
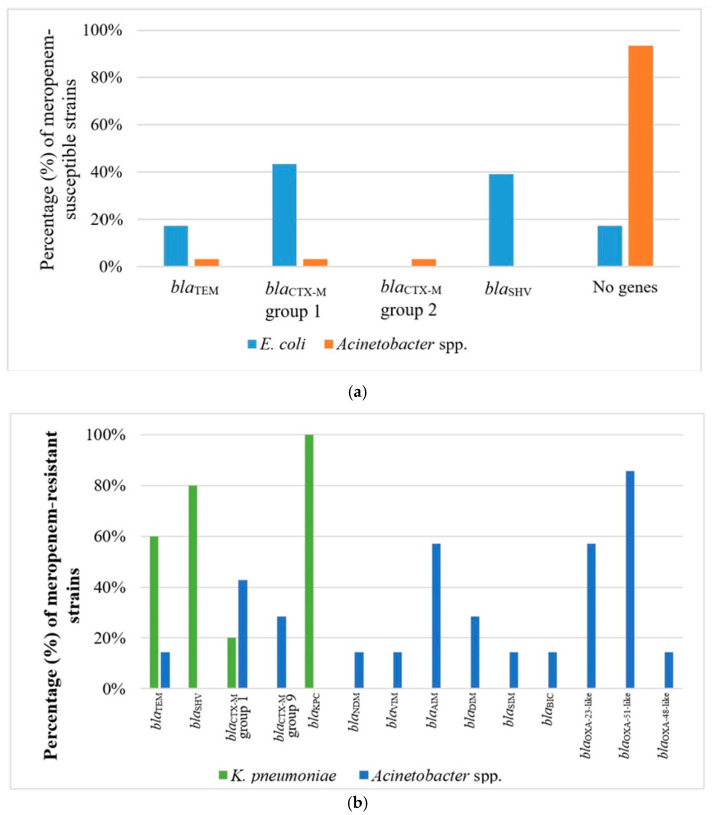
Percentage of *E. coli, K. pneumoniae,* and *Acinetobacter* strains in which β-lactamase genes were detected; (**a**) meropenem-susceptible strains, (**b**) meropenem-resistant strains.

All identified *E. coli* and *K. pneumoniae* strains resistant to β-lactams were phenotypically confirmed as ESBL and carbapenemase producers, respectively. The molecular screening of β-lactamase genes showed that nine *E. coli* strains harbored *bla*_SHV_ genes alone (39.1%), six harbored *bla*_CTX-M_ genes of group 1 (26.1%), and four harbored both *bla*_TEM_ and *bla*_CTX-M_ genes of group 1 (17.4%). In four *E. coli* strains (17.3%), no β-lactamase genes were detected (Figure 3, Table S1). Regarding the non-clinical *K. pneumoniae* strains, three strains harbored *bla*_TEM,_ *bla*_SHV_, and *bla*_KPC_ genes (60.0%); one strain carried *bla*_SHV_, *bla*_CTX-M_ genes of group 1, and *bla*_KPC_ genes (20.0%); and one strain harbored *bla*_KPC_ alone (Figure 3, Table S2). The clinical *K. pneumoniae* strains harbored a broader range of β-lactamase genes compared to the non-clinical ones, ranging from two (21.1%, n = 4) to seven (15.8%, n = 3) different β-lactamase genes. The ESBL genes detected were *bla*_SHV_ (89.5%, n = 17), *bla*_CTX-M_ genes of group 1 (89.5%, n = 17), *bla*_TEM_ (57.9%, n = 11), *bla*_VEB_ (57.9%, n = 11), *bla*_PER_ (31.6%, n = 6), and *bla*_OXA-1_ (36.8%, n = 7), whereas no AmpC genes were detected. Concerning the carbapenemase genes, *bla*_KPC_ (26.3%, n = 5), *bla*_NDM_ (73.7%, n = 14), and *bla*_OXA-48_ (31.6%, n = 6) were identified. In two carbapenem-resistant clinical strains, no carbapenemase genes were identified, but harbored *bla*_OXA-1_ and *bla*_CTX-M_ genes of group 1.

Among the 31 non-clinical meropenem-susceptible *Acinetobacter* strains, only two (6.5%) were found to carry a β-lactamase gene. Specifically, one of these strains carried *bla*_TEM_ alone, and the other harbored *bla*_CTX-M_ genes of groups 1 and 2. However, all the non-clinical meropenem-resistant strains carried β-lactamase genes, ranging from one (two strains, 28.6%, n = 2) to six distinct genes (42.9%, n = 3). More specifically, the genes *bla*_TEM_, *bla*_BIC_, *bla*_SIM_, *bla*_VIM_, *bla*_NDM_, and *bla*_OXA-48_ were detected in one strain each (14.3%). Other genes that were found were *bla*_CTX-M_ genes of group 1 (42.9%, n = 3) and 9 (28.6%, n = 2), *bla*_AIM_ (57.1%, n = 4), *bla*_DIM_ (28.6%, n = 2), *bla*_OXA-23_ (57.1%, n = 4), and *bla*_OXA-51_ (85.7%, n = 6) (Figure 3, Table S3). The clinical *Acinetobacter* strains also carried two (43.75%, n = 7) to seven (6.25%, n = 1) different β-lactamase genes. The ESBL genes detected were *bla*_TEM_ (25%, n = 4), *bla*_CTX-M_ genes of group 1 (6.25%, n = 1), and *bla*_CTX-M_ genes of group 9 (6.25%, n = 1), whereas no AmpC genes were detected. Concerning the carbapenemase genes, *bla*_OXA-23_ and *bla*_OXA-51_ were identified in all clinical strains (100%). Other carbapenemase genes detected were *bla*_VIM_ (37.5%, n = 6), *bla*_DIM_ (18.75%, n = 3), and *bla*_AIM_ (12.5%, n = 2).

### 2.4. Proteomic Relationship of Strains

**Figure 4:**
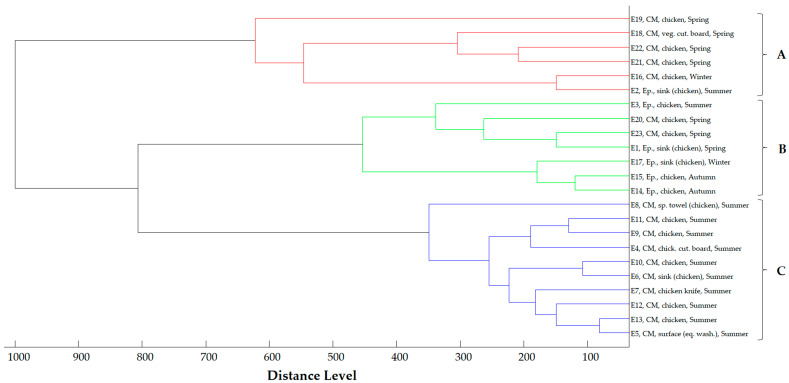
Main spectra dendrogram of *E. coli* strains (strain I.D., origin and season of isolation, CM = Central Macedonia, Ep. = Epirus). Clusters are defined by capital letters A, B and C.

The main spectra dendrogram of the *E. coli* strains (Figure 4) revealed three distinct clusters at a cut-off value of 400 distance units. Cluster A includes six strains—five from the Central Macedonia Region and one from the Epirus Region—isolated from chicken and the hospital kitchen environment, primarily in the summer and secondarily in autumn. Cluster B comprises seven strains isolated across all geographical regions and seasons, while Cluster C consists of 10 strains recovered solely from the Central Macedonia Region in the summer.

**Figure 5:**
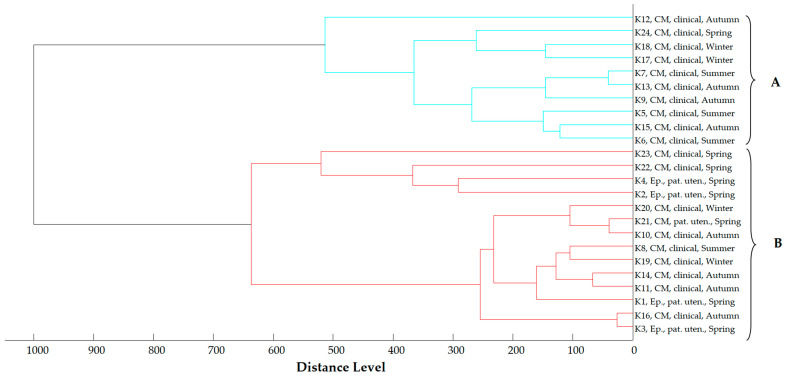
Main spectra dendrogram of *K. pneumoniae* strains (strain I.D., origin and season of isolation, CM = Central Macedonia, Ep. = Epirus). Clusters are defined by capital letters A and B.

In the main spectra dendrogram of *K. pneumoniae* strains (Figure 5), the strains are organized into two clusters. Cluster A includes 10 clinical strains isolated during all seasons, whereas Cluster B consists of both clinical and environmental strains collected across all geographical regions and seasons.

**Figure 6:**
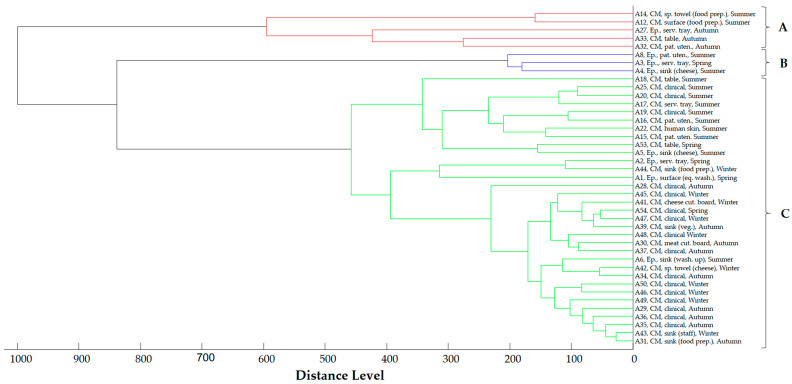
Main spectra dendrogram of *A. baumannii* strains (strain I.D., origin and season of isolation, CM = Central Macedonia, Ep. = Epirus). Clusters are defined by capital letters A, B and C.

Similarly, the main spectra dendrogram of *Acinetobacter* spp. (Figure 6) shows three clusters. Cluster A consists of four strains, primarily isolated from the hospital kitchen environment in the Epirus Region during the summer and autumn. Cluster B includes three strains isolated from the hospital kitchen environment in the Epirus Region during the summer and spring. Cluster C encompasses both clinical and non-clinical strains recovered across all geographical regions and seasons.

In all three dendrograms, a close relationship is mainly observed among strains collected within the same Region and season. However, close relationships are also evident among strains isolated from different seasons and geographical regions. Additionally, close relationships were noted between specific food and environmental strains, as well as between clinical and environmental strains. 

### 2.5. Phylogenetic Analysis of E. coli Strains

In the phylogenetic analysis of the *E. coli* strains, the majority were classified into phylogenetic groups A (73.9%, n = 17) and B1 (17.4%, n = 4). However, two strains (8.7%) did not display any of the analyzed genes, resulting in their classification as of an unknown phylogenetic group (Table S1).

### 2.6. Medical History Results

Resistant strains were identified in a 63-year-old male chef from the hospital in the Central Macedonia Region. The chef is a non-smoker but consumes a significant amount of alcohol, averaging one glass of wine daily. He suffers from asthma and nasal turbinate hypertrophy and is allergic to Brazilian nuts. He has not undergone recent surgery or hospitalization and has not taken antibiotics close to the sampling period. However, he keeps a stock of antibiotics at home for emergency. Among the other 24 hospital employees from whom no resistant strains were isolated, 14 have smoked at least once (10 of them are current smokers), and nine abstain from alcohol. Half of these employees have at least one health condition, with two recently hospitalized due to immunosuppressive treatment. Additionally, two underwent recent surgical procedures, and half received antibiotics close to the sampling period. No significant differences were observed in human characteristics and behavior regarding antibiotic use between hospital staff with positive and negative test results (Tables S4–S7).

## 3. Discussion

Antimicrobial resistance is a critical public health emergency that limits therapeutic options, particularly for nosocomial infections. Of particular concern are Gram-negative bacteria, such as *E. coli*, *K. pneumoniae*, and *A. baumannii*, which are commonly isolated from hospitalized patients and exhibit resistance to critically important antimicrobials, including third-, fourth-, and fifth-generation cephalosporins and carbapenems [20]. Animals also serve as reservoirs for these resistant pathogens, as inappropriate antibiotic usage in veterinary medicine promotes the emergence of resistant bacteria and facilitates the transmission of AMR mobile genetic elements among bacterial pathogens [21]. Hospital kitchens, responsible for preparing patients’ meals, receive large quantities of various food products, including poultry meat, frequently harboring resistant bacteria [22]. Consequently, hospital kitchens are potential reservoirs for transmitting resistant strains from food products to the environment, kitchen employees, or patients, thereby contributing to the circulation of resistant strains within the hospital.

In this study, 35.0% of chicken samples and 4.5% of environmental samples (sinks, surfaces, equipment) from hospital kitchens were tested positive for ESBL *E. coli*. Notably, all but one of the positive environmental samples had a direct connection to chicken. This aligns with the findings of Tschudin-Sutter et al. [19], who reported a 6.5% prevalence of ESBL *E. coli* in chicken cutting boards from a hospital kitchen in Switzerland (n = 10). Kitchens are generally considered reservoirs of human pathogens, especially when unsafe food preparation and storage behaviors are performed, due to favorable conditions for the survival and growth of microorganisms, such as high humidity levels and nutrients from food [23,24]. Sinks especially are frequently contaminated sites in kitchens, harboring foodborne pathogens, such as *E. coli* [23]. El-Liethy et al. [24] found the highest bacterial counts in biofilm samples from kitchen sink drainage pipes, while the toilet area showed little contamination from pathogenic microorganisms and fecal-origin microbes, with *E. coli* being the most prevalent microorganism in all biofilm samples. Poultry is a significant reservoir of *E. coli* strains among food commodities, including β-lactam-resistant strains [22], and improper handling of poultry can disseminate ESBL *E. coli* strains into the surrounding environment, as evidenced by this study. Yet, varying prevalence of ESBL *E. coli* was observed in raw chicken from hospital kitchens. Stewardson et al. [25] reported that 86% (n = 30) of raw chicken samples from hospital kitchens in Switzerland were positive for ESBL *E. coli*. Conversely, Gómez-Sanz et al. [26] found that only 17.6% (n = 83) of chicken samples from large supermarket chains, small local food retailers, and the University Hospital Basel kitchen in Switzerland tested positive for ESBL *E. coli*. They observed no significant differences in the occurrence of ESBL *Enterobacteriaceae* between shop and hospital kitchen samples, with chicken being the most commonly contaminated food product (25.9%), followed by sprouts (15.2%). These disparities in prevalence rates can be attributed to differences in sampling techniques, sample sizes, and methodologies, as well as variations in microbial population dynamics in poultry meat based on geographical location or country [18,27].

The observed prevalence of *K. pneumoniae* and *Acinetobacter* spp. in the hospital kitchen environment samples collected was 1.5% and 15.0%, respectively. Dramowski et al. [28] reported that, among 100 surface and equipment swabs in a neonatal ward in South Africa, including kitchen areas, three strains of *Acinetobacter* spp. and one of *K. pneumoniae* were detected, with a predominance of Gram-negative pathogens on moist surfaces and equipment. In this study, *K. pneumoniae* was isolated solely from utensils used by hospital patients, suggesting that contamination occurred in the wards rather than the kitchen, as the utensils were returned to the kitchen for washing. This finding aligns with Calbo et al. [29], who highlighted the role of hospital kitchens in distributing ESBL *K. pneumoniae* during a foodborne nosocomial outbreak in Spain. *Acinetobacter* spp. was isolated from a broader range of kitchen samples, including utensils used by patients, serving trays, tables, sinks, and surfaces. Among environmental samples, sponges and sponge towels frequently harbored *Acinetobacter* spp. and *E. coli* strains. Kitchen sponges, sponge towels, and dishcloths have been demonstrated to serve as reservoirs and disseminators of pathogenic microorganisms in consumer kitchens, especially species of the *Acinetobacter* genus [23,30]. Positive environmental samples were mainly associated with cheese, vegetables, red meat, food preparation, washing up, and personnel hygiene. Araújo et al. [31] similarly reported resistant *Acinetobacter* spp. in infant milk formula and utensils used for the preparation and distribution of the formula in a nursery room of a public hospital in Brazil. Interestingly, no *Acinetobacter* spp. or *K. pneumoniae* strains were found in chicken samples or related environmental samples. Gómez-Sanz et al. [26] noted that only 1% (n = 5) of chicken samples from shops and a hospital kitchen in Switzerland tested positive for ESBL *K. pneumoniae*, with sprouts showing higher contamination (12.1%). Similarly, Stewardson et al. [25] stated that 3% (n = 2) of chicken samples from supermarkets and a hospital in Switzerland were positive for *K. pneumoniae*. *Acinetobacter* spp. have also been described as common saprophytic microorganisms of poultry meat [17].

Seasonal and regional differences in the prevalence of resistant strains were also observed in this study. The prevalence of *E. coli* in the hospital kitchen environment was significantly higher in the summer compared to other seasons, whereas all *K. pneumoniae* strains were found in the spring. These disparities may be attributed to lapses in hygienic practices during the warmer months. Ranjbar et al. [32] reported a high incidence of *E. coli* strains in hospital food samples during the summer, potentially linked to lower levels of individual hygiene in this season. In our study, no significant differences were observed in the prevalence rates of *E. coli* in chicken samples across seasons. However, a slightly higher isolation rate was noted in the summer and spring. These results align with Gómez-Sanz et al. [26], who found no monthly differences in the rate of ESBL-*Enterobacteriaceae*-positive samples over a two-year period in shops and a hospital in Switzerland. Yousif et al. [33] also observed higher total aerobic counts and populations of coliforms and *S. aureus* in chicken samples from a hospital kitchen in Egypt during summer compared to winter, though no significant differences were observed for *E. coli* populations throughout the seasons. It is noteworthy that *E. coli* and *K. pneumoniae* infection rates have been found to peak in the summer [34,35]. Consequently, elevated temperatures and increased antibiotic usage to manage prevalent summer infections could partially explain the heightened prevalence rates of ESBL/AmpC *E. coli* in the hospital environment during warmer seasons. Regarding regional variations, a higher prevalence of resistant *Acinetobacter* spp. was observed in the hospital kitchen environment in Central Macedonia compared to the Epirus Region. Additionally, higher isolation rates of ESBL *E. coli* were observed in chicken samples from Central Macedonia. The Region’s lowland terrain contrasts with Epirus’s mountainous Region, and these different topographies are associated with varying regional climate conditions, potentially affecting the microbial populations in food samples from each Region [36]. Differences in the prevalence of resistant bacteria in poultry meat across various geographical regions within the same country have been reported in other studies [37].

In the present study, only one skin sample tested positive for β-lactam-resistant *Acinetobacter* spp. (4.0%), isolated from the hands of one chef working in the hospital of the Central Macedonia Region, where the prevalence of *Acinetobacter* spp. in the kitchen was significantly higher. Furthermore, the positive sample was collected during summer, when slightly higher populations of *Acinetobacter* spp. were observed in the kitchen environment. These factors, coupled with the absence of *Acinetobacter* spp. strains from hospital staff during other seasons or in the other hospital, suggest that the increased prevalence of *Acinetobacter* spp. in the environment may pose a potential risk for transmission to kitchen employees when good hygiene practices are not strictly followed. Morgan et al. [38] reported that 38.7% (n = 77) of interactions between healthcare workers and patients colonized by MDR *A. baumannii* in a hospital in Maryland (Baltimore, USA) resulted in workers’ contamination of gloves and/or gowns, while 4.5% of interactions resulted in contamination of workers’ hands after glove removal and before hand hygiene. Similarly, Bayuga et al. [39] found that 3.3% (n = 6) of healthcare workers in two hospitals in New York City (USA) tested positive for *A. baumannii* on their hands and/or nares. In this study, no resistant strains were recovered from oropharynx samples. However, it has been documented that throat colonization by *Acinetobacter* spp. can occur in up to 10% of community members who consume excessive amounts of alcohol [40], including both resistant and sensitive variants of *A. baumannii*, which might explain the discrepancies observed in our results.

Additionally, no resistant *E. coli* and *K. pneumoniae* strains were found in skin samples. The isolation of these bacteria has been reported from the hospital personnel in several countries, such as Switzerland [19,25], Spain [29], and the USA [41]. The samples collected in this study, where no rectal swabs or fecal samples were taken, may explain the lower prevalence observed, as rectal swabs are considered more sensitive but often have low compliance. However, Bitterman et al. [42] report that none of the 177 healthcare workers sampled from a hospital in Israel were fecal carriers of carbapenem-resistant *Enterobacteriaceae*. The lower prevalence of carbapenem-resistant strains compared to ESBL strains can be attributed to the less frequent use of carbapenems compared to cephalosporins, as carbapenems are considered critically important antimicrobials used only as last-resort options [20], and most strains remain susceptible to carbapenems. The differences in prevalence compared to this study can be explained by the fact that those strains included both susceptible and resistant strains and that sampling was performed on medical staff rather than food handlers, who do not have direct contact with patients.

Regarding the antimicrobial susceptibility of the strains, all *E. coli* strains were MDR and phenotypically confirmed as ESBL-producers, whereas all non-clinical *K. pneumoniae* strains were MDR and phenotypically confirmed as presumptive carbapenemase producers. Specifically, all *E. coli* strains demonstrated resistance to CAZ, CTX, and AM, with particularly high resistance rates observed for TE, FEP, and TPZ. Similarly, all non-clinical *K. pneumoniae* strains showed resistance to most antimicrobials tested. The resistance rates of *E. coli* and *K. pneumoniae* observed in this study were higher compared to other studies from Switzerland [25] and Spain [26,29]. On the other hand, Ranjbar et al. [32] reported that all *E. coli* strains found in hospital food samples in Iran were MDR, with resistance against 10, 11, 12, and more than 12 antibiotics at 37.5%, 25.0%, 18.8%, and 12.5%, respectively. The highest resistance levels were observed for ampicillin (93.75%), gentamycin (93.75%), tetracycline (87.50%), ciprofloxacin (81.25%), and amikacin (75%). The discrepancies observed with the results of this study can be attributed to the selective enrichment and isolation using β-lactams, positively affecting the isolation of β-lactam-resistant and MDR strains. Additionally, Greece is among the European countries with the highest AMR rates in hospitals [15], which likely explains the recovery of strains with elevated resistance rates.

All *E. coli* strains were susceptible to carbapenems and colistin, and most *K. pneumoniae* strains were susceptible to colistin. Regarding non-clinical *Acinetobacter* spp., although most meropenem-susceptible strains were classified as MDR, they generally showed susceptibility to many antimicrobial classes, with most strains exhibiting resistance to CAZ, CTX, PRL, and TPZ. Conversely, all meropenem-resistant *Acinetobacter* spp. strains were characterized as XDR and exhibited resistance to almost all antimicrobials tested. The results of this study align with other studies. Araújo et al. [31] reported that most *Acinetobacter* spp. (82.35%, n = 14) isolated from infant milk formula and utensils in a hospital in Brazil were classified as MDR. The highest resistance rates were observed for ampicillin-sulbactam (88.2%), cefotaxime (82.3%), and trimethoprim-sulfamethoxazole (70.6%), with one MDR strain from a utensil (jar) also resistant to imipenem. Bayuga et al. [39] report that only one out of six *Acinetobacter* strains recovered from healthcare workers in two hospitals in the USA was classified as MDR. Similar to *E. coli*, all non-clinical *Acinetobacter* strains in the present study exhibited susceptibility to colistin. However, a significant number of clinical *A. baumannii* and *K. pneumoniae* strains exhibited resistance to colistin. More specifically, clinical strains showed resistance to most antimicrobials tested, leading to the classification of most strains as MDR or XDR. Additionally, 43.8% of *A. baumannii* strains (n = 7) were classified as PDR. The resistance profiles and rates observed align with most Greek studies characterizing AMR clinical strains in Greek hospitals [43,44]. In *K. pneumoniae* and meropenem-susceptible *Acinetobacter* strains, no common resistance profiles were observed between clinical and non-clinical strains. However, the majority of non-clinical meropenem-resistant *Acinetobacter* strains shared the same resistance profile as XDR clinical strains, which were only susceptible to colistin. Gong et al. [45] reported that clinical *A. baumannii* strains from a burn intensive care unit (ICU) in a Chinese hospital exhibited resistance to more antimicrobials than strains from the ward environment. Conversely, Hu et al. [46] found that environmental strains of carbapenem-resistant *A. baumannii* in an ICU of another Chinese hospital had similar resistance profiles to clinical ones. Similar resistant profiles were observed in carbapenem-resistant Enterobacterales between environmental and clinical strains in three hospitals in Korea, with environmental strains generally resistant to almost all antimicrobial agents tested except amikacin [47]. It is important to note that in previous studies, environmental samples were taken from clinical wards in close vicinity to patients, unlike the hospital kitchens sampled in the present study. Nonetheless, most of our results are consistent with the findings of those studies.

Regarding β-lactamase genes, *bla*_CTX-M_ of group 1 was the predominant ESBL gene in *E. coli* strains, closely followed by *bla*_SHV_, while *bla*_TEM_ genes were identified in fewer strains. The *bla*_CTX-M_ genes are among the most commonly isolated ESBL genes in Enterobacterales, with the most commonly detected variants including CTX-M-14 (group 9) and CTX-M-1 (group 1) among animal strains, as well as CTX-M-15 (group 1) among human strains [21]. Tschudin-Sutter et al. [19] reported that *bla*_CTX-M-1_ was the most prevalent ESBL gene in *E. coli* strains from chicken cutting boards and gloves of kitchen personnel after handling poultry in a hospital in Switzerland, followed by *bla*_SHV-12_, with *bla*_CTX-M-14_ also identified in one *E. coli* strain from a chicken cutting board. In another hospital in Switzerland, *bla*_CTX-M_ genes were the most frequently identified genes among ESBL *Enterobacteriaceae*, followed by *bla*_TEM_ and *bla*_SHV-12_ genes [25]. Food strains more commonly possessed *bla*_CTX-M-1_, whereas *bla*_CTX-M-14_ and *bla*_CTX-M-15_ were predominant among strains of human (food handler or patient) origin [25]. SHV variants were also prevalent in *E. coli* strains from chicken samples collected from a hospital in Iran [32]. In a foodborne nosocomial outbreak in Spain, the *K. pneumoniae* strains responsible for the outbreak and isolated from food samples, the kitchen environment, and kitchen employees harbored *bla*_SHV-1_ and *bla*_CTX-M-15_ [29]. Moreover, in this study, all non-clinical *K. pneumoniae* strains harbored the KPC carbapenemase, which was the only carbapenemase identified in these strains. Clinical *K. pneumoniae* strains harbored more β-lactamase genes compared to the non-clinical ones. Besides the genes found in non-clinical strains, clinical strains additionally possessed ESBL genes *bla*_VEB_, *bla*_PER_, and *bla*_OXA-1_, as well as carbapenemase genes *bla*_NDM_ and *bla*_OXA-48_. The identified genes in the clinical strains have also been reported in other clinical *K. pneumoniae* strains by other studies from Greece [43]. In two carbapenem-resistant clinical strains, no carbapenemase genes were identified, as they only harbored *bla*_OXA-1_ and *bla*_CTX-M_ group 1 genes. These strains may possess other carbapenemases not investigated in this study, or they might acquire resistance through other mechanisms such as ESBL hyper-production, decreased outer membrane permeability due to porin alteration or loss, and efflux pump overexpression [48]. Regarding *Acinetobacter* spp., most meropenem-susceptible strains did not harbor the investigated β-lactamase genes, although a few carried *bla*_TEM_ and *bla*_CTX-M_ genes of group 1 or 2. Similar to carbapenem resistance, cephalosporin resistance in Gram-negative bacteria, including *E. coli*, *K. pneumoniae*, and *A. baumannii*, can be attributed to various other mechanisms, such as target alteration of penicillin-binding proteins (PBPs), decreased permeability of β-lactams into the bacterial cell, and efflux pump upregulation [49]. Additionally, *Acinetobacter* strains often carry an AmpC cephalosporinase known as Acinetobacter-derived cephalosporinase (ADC), which confers resistance to extended-spectrum cephalosporins [50]. In contrast to meropenem-susceptible strains, all non-clinical meropenem-resistant *Acinetobacter* strains harbored β-lactamase genes. Specifically, *bla*_CTX-M_ genes of group 1 predominated among the ESBL genes, followed by *bla*_CTX-M_ genes of group 9 and *bla*_TEM_ variants. The most commonly identified carbapenemase gene was *bla*_OXA-51_, followed by *bla*_OXA-23_, *bla*_AIM_, *bla*_DIM_, *bla*_SIM_, *bla*_BIC_, *bla*_NDM_, *bla*_VIM_, and *bla*_OXA-48_. No *Acinetobacter* strain harbored a *bla*_KPC_ carbapenemase-encoding gene. OXA-type carbapenemases, such as OXA-23, OXA-24, and OXA-58, are frequently acquired and expressed by carbapenem-resistant *Acinetobacter* spp., while OXA-51 appears to be intrinsic to *A. baumannii* [51]. Moreover, four types of Ambler Class B carbapenemases (metallo-β-lactamases, MBLs) have been described in *A. baumannii*: IMP, VIM, NDM, and SIM [52]. AIM, DIM, and BIC enzymes are acquired carbapenemases generally considered “minor carbapenemases”, primarily identified in *Pseudomonas aeruginosa*, *Pseudomonas stutzeri*, and *Pseudomonas fluorescens*, respectively [53,54]. To the best of our knowledge, this study is likely the first to report the identification of these minor carbapenemases in carbapenem-resistant *Acinetobacter* strains from Greek hospitals. Chen et al. [55] reported similar detection rates of *bla*_AIM-1_ (43.38%, n = 59) in imipenem-resistant *A. baumannii* strains from patients in a hospital in China. However, most previous studies searching for these carbapenemases in *Acinetobacter* strains reported negative results [56,57]. In this study, *bla*_AIM_ and *bla*_DIM_ carbapenemases were also found in clinical strains, which, in contrast to *K. pneumoniae* strains, generally showed a similar distribution of β-lactamase genes compared to non-clinical carbapenem-resistant *Acinetobacter* strains. The acquisition of minor carbapenemases by *Acinetobacter* strains can be attributed to the horizontal transfer of these genes from resistant *Pseudomonas* spp., as carbapenem-MDR *Pseudomonas* spp. are endemic in both hospitals sampled in this study, based on information shared by the infectious diseases department of those hospitals.

Based on the protein spectra of the strains, three distinct clusters were identified for *E. coli* and *A. baumannii* strains, while *K. pneumoniae* strains formed two distinct clusters. The distribution of the strains into different clusters seems to be influenced by the geographical origin and seasonality. In all three microbial species, a close relationship was frequently observed among strains collected within the same hospital and season. Furthermore, the close relatedness observed among strains from different environmental samples within the same hospital hints at potential cross-contamination and circulation of resistant microorganisms in the kitchen environment. Moreover, certain strains found during different seasons displayed close genetic relatedness, underscoring the persistent nature of resistant strains in the environment [40,58,59]. Close relationships were also observed among strains recovered from chicken and environmental samples that came in contact with it, suggesting that poultry represents a vehicle for the import of resistant strains from the outside environment to the hospital kitchen. Similarly, small distance levels were observed among clinical and environmental strains, especially utensils previously used by patients and serving trays, implying that this equipment was contaminated by hospital patients in the wards and subsequently transferred to the kitchen. The entrance of resistant strains into the kitchen through various vehicles, such as food and equipment used by patients, highlights the significant role of the kitchen in the circulation and dissemination of these strains in the hospital environment. Benbow et al. [60] stated that in a nosocomial outbreak of NDM *Enterobacter cloacae* and *E. coli* in two hospitals in the United Kingdom, electric floor scrubbers used for cleaning the hospital catering facilities and associated toilets may have contributed to the dissemination of resistant bacteria in the hospital environment. Calbo et al. [29] also mentioned that in a nosocomial outbreak in Spain, genotypic analysis of all clinical, environmental, and fecal carrier strains showed the dissemination of a single strain of ESBL *K. pneumoniae*, providing evidence that food prepared in the hospital kitchen can be a transmission vector for ESBL *K. pneumoniae*. On the other hand, Stewardson et al. [25] stated that the *E. coli* strains derived from humans (patients, food handlers) and food samples in a hospital in Switzerland were largely distinct. In this study, the chef’s *A. baumannii* strain exhibited a closer relationship with clinical strains and strains recovered from utensils used by patients. Kitchen staff is a group of hospital employees at higher risk of exposure to resistant bacteria; measures should be taken to prevent transmission and colonization of kitchen staff with resistant bacteria.

Regarding the phylogenetic analysis of *E. coli* strains, the majority clustered into phylogenetic group A, with fewer assigned to group B1, while no strains were classified into groups D, E, C, F, B2, and clade I, suggesting that the *E. coli* strains recovered are primarily commensal. However, phylogroups A, B1, and C may also include intestinal pathogenic *E. coli* responsible for dysentery and hemolytic uremic syndrome, whereas phylogenetic groups B2, D, E, and F are associated with extra-intestinal pathogenic strains [61,62]. Additionally, two strains had an unidentified phylogenetic group. No differences were observed in the distribution of phylogenetic groups of *E. coli* strains among samples, as phylogroup A predominated in both chicken and environmental samples, while *E. coli* strains of phylogroup B1 were also recovered from both types of samples. The discrepancies observed in the results of different studies underscore how prevailing conditions, influenced by geographical region, season, and type of sample, can affect the selection and predominance of specific phylogenetic groups of *E. coli* in food and the hospital kitchen environment.

In this study, an effort was made to investigate potential factors and behaviors contributing to the isolation of resistant strains from kitchen staff. No significant differences were observed between positive-tested and negative-tested kitchen staff regarding their demographic characteristics, health status, and behavior towards antibiotic use, despite the fact that 12 (48%) employees received antibiotics at least once close to the sampling period. Interestingly, the chef tested positive for *Acinetobacter* spp. in the hospital and season where the highest prevalence of *Acinetobacter* spp. in the kitchen environment was observed, and closer relationships were noted among the chef’s strains and those recovered from utensils previously used by patients. Similar results were also mentioned by other researchers [25,39]. The implementation of strict hygiene practices in hospital settings and kitchens and the education of all hospital staff, including kitchen employees, on the importance of AMR and how resistant strains spread in healthcare environments, particularly to prevent cross-contamination when interacting with patients, are some measures that could be implemented to control the circulation of resistant strains between the hospital kitchens and wards. Additionally, regular monitoring of significant resistant strains in both kitchen and hospital environments is of primary importance.

## 4. Materials and Methods

### 4.1. Sampling

This study was conducted in two hospitals located in the Regions of Central Macedonia and Epirus. The hospital in Central Macedonia is medium-sized (175 beds), while the hospital in the Epirus Region is considered large (760 beds). Sampling was performed in the kitchen areas of the hospitals, including the collection of environmental samples, chicken samples, and samples from hospital staff. Sampling was conducted each season from spring 2023 to winter 2024 to explore any seasonal variations. A total of 320 samples were collected, with 40 samples from each hospital in each season (25 environmental samples, 5 chicken samples, 10 human samples).

A total of 200 samples were collected from the environment of hospital kitchens. Specifically, 40 samples were collected from surfaces, 40 from equipment, 40 from sinks, 40 from utensils used by hospital patients (cutlery, dishes, and glasses), 24 from serving trays, and 16 from tables used by hospital staff (Table S8). Environmental samples were collected using sterilized gauzes or swabs, saturated with 5 mL of Buffered Peptone Water (BPW, Oxoid, Basingstoke, UK), which were used to wipe a 100 cm² surface area. The swabs or gauzes were then placed in test tubes containing 5 mL BPW. The sponge towels and sponges were collected aseptically and placed in sterile containers. Additionally, 40 chicken breast samples were collected aseptically from the hospital kitchens using sterile utensils. All samples were transported under refrigeration (<4 °C) to the Laboratory of Animal Food Products Hygiene and Veterinary Public Health within 24 h of collection for further analysis.

Furthermore, 80 samples were collected from hospital staff working in the kitchen or in close proximity (chefs, food service workers, nurses, cleaning staff). Specifically, 40 samples each were taken from the skin (hands and arms) and the oropharynx of 25 hospital employees. For skin sampling, sterile swabs soaked in 5 mL BPW, along with dry sterile swabs, were used to wipe the entire surface area of both hands. Oropharynx samples were obtained using sterile swabs, which were placed in test tubes containing 5 mL BPW. Before sampling, the hospital staff were asked to answer questions regarding their medical history and antibiotic consumption in order to identify potential factors, practices, and behaviors contributing to the isolation of resistant strains from humans (Table S9).

Additionally, 19 *K. pneumoniae* and 16 *A. baumannii* strains previously isolated from hospitalized patients were included in the study. These strains were obtained from patients in the Intensive Care Unit (16 *K. pneumoniae* and 14 *A. baumannii* strains), the Internal Medicine Clinic (3 *K. pneumoniae* strains), and the Orthopedics Clinic (2 *A. baumannii* strains) of the hospital in Central Macedonia (Table S10). The inclusion of these strains aimed to investigate potential relationships between the clinical strains and those recovered from the environment of hospital kitchens and staff.

### 4.2. Microbiological Examination, Identification, and Proteomic Relationship of the Isolated Strains

The experimental protocol aimed to selectively isolate resistant bacteria from the collected samples. To isolate strains producing ESBL, AmpC, and carbapenemase, the methods outlined by EFSA (2011; 2013) [48,63] and Carvalheira et al. [64] were used, with modifications. Sponge towels, sponges, and 25 g of chicken breast were initially placed into stomacher bags containing sterile Buffered-Peptone Water (BPW, Oxoid, Basingstoke, UK) and homogenized using a stomacher (Interscience, Saint Nom la Bretêche, France) for 2 min. Subsequently, the stomacher bags and the test tubes with swabs from environmental and human samples were incubated at 37 °C for 2 h as a pre-enrichment process to facilitate the recovery of microorganisms from any cellular damage. After incubation, 100 μL of the rinsates were plated onto McConkey or Dijkshoorn broth (Oxoid, Basingstoke, UK) supplemented with either 1 mg/L ceftazidime (ceftazidime sodium salt, Sigma-Aldrich, St. Louis, MI, USA) or 0.125 mg/L meropenem (meropenem sodium salt, Sigma-Aldrich, USA). McConkey broth with antibiotics was used for isolating resistant *E. coli*, *K. pneumoniae*, and *Acinetobacter* spp., while Dijkshoorn broth served as an additional selective enrichment medium for resistant *Acinetobacter* spp. [64]. The McConkey broth cultures were incubated at 37 °C for 24 h, whereas the Dijkshoorn broth cultures were incubated at 30 °C for 24–48 h. After enrichment, 10 μL of McConkey broth cultures were surface-inoculated onto Chromocult TBX agar (Merck GmbH, Darmstadt, Germany) and McConkey agar (Oxoid, Basingstoke, UK) supplemented with 1 mg/L ceftazidime or 0.125 mg/L meropenem, followed by incubation at 44 °C and 37 °C, respectively, for 24 h. Similarly, CHROMagar™ Acinetobacter (CHROMagar, Saint-Denis, France) supplemented with 1 mg/L ceftazidime or 0.125 mg/L meropenem was inoculated with 10 μL of the Dijkshoorn broth cultures and incubated at 30 °C for 24–48 h. *E. coli* forms blue to green colonies on Chromocult TBX agar, whereas *K. pneumoniae* typically forms red to pink mucoid colonies on McConkey agar. Red colonies on CHROMagar Acinetobacter, as well as colorless to light pink colonies on McConkey agar, were considered typical of *Acinetobacter* spp. Up to five characteristic colonies from each plate were selected and sub-cultured on Tryptic Soy Agar (TSA, Oxoid, Basingstoke, UK).

The identification of isolated strains was conducted using matrix-assisted laser desorption/ionization (MALDI) coupled with a time-of-flight (TOF) mass spectrometry analyzer (MALDI–TOF MS). This analysis was performed on a Microflex LT mass spectrometer (Bruker Daltonics, Bremen, Germany). Characteristic colonies of *E. coli*, *K. pneumoniae*, and *Acinetobacter* spp. grown on TSA were chosen for protein extraction using the formic acid method, following the manufacturer’s protocols. A single colony of each strain was transferred into an Eppendorf tube containing 300 μL of ultrapure water, homogenized, and mixed with 900 μL of pure ethanol. The tubes were then homogenized, centrifuged at 13,000 rpm for 2 min, and the supernatant was removed. This step was repeated once more, and the tubes were left open for 5 min. After ethanol evaporation, 30 μL of 70% formic acid and 30 μL of acetonitrile were added to the protein extract and thoroughly mixed. The mixture was then centrifuged at 13,000 rpm for 2 min, and 1 μL of the protein extract was applied to the MALDI–TOF MS target plate. Each spot on the plate was overlaid with 1 μL of matrix solution [a saturated solution of cyano-4-hydroxycinnamic acid matrix (Bruker Daltonics) in 50% acetonitrile (SigmaAldrich, St. Louis, MO, USA) with 25% trifluoroacetic acid (SigmaAldrich, USA)] and allowed to air-dry. Protein profiles were obtained using linear positive mode analysis with a laser frequency of 20 Hz, and raw protein spectra were automatically collected over a mass range of 2000–20,000 Da using the AutoXecute control software (Flex control 3.4; Bruker Daltonics). Calibration was performed using the Bruker Bacterial Test Standard (BTS). Strain identification was carried out using MALDI Biotyper software version 4.0, and the collected spectra were compared with those in the mass-spectrum library (v6.093 MSPs). Results were categorized based on adjusted score values, according to the manufacturer’s recommendations. A dendrogram of main spectra (MSP) was generated to cluster and explore relationships among isolated strains. Each spectrum underwent smoothing and baseline subtraction using the MALDI Biotyper Offline Classification 4.0 software with default parameters. The MSP dendrogram was created with a cutoff value set at 400 distance level for optimal discriminatory power, as proposed by Peratikos et al. [65], with modifications. Recovered strains of *E. coli*, *K. pneumoniae*, and *Acinetobacter* spp. of the ACB complex were preserved at −80 °C after addition of 15% glycerol until further analysis.

### 4.3. Antimicrobial Susceptibility Testing of the Isolated Strains

The susceptibility of isolated strains to antibiotics was assessed using the disk diffusion method, following the Clinical and Laboratory Standards Institute guidelines [66]. From an overnight culture a 0.5 McFarland suspension adjusted with a nephelometer (Biosan, Rīga, Latvia) was streaked onto Mueller Hinton agar plates (bioMérieux, Marcy l’Etoile, France), the antibiotic disks were placed and the plates were incubated at 35 °C for 24 h. The susceptibility of *E. coli/K. pneumoniae* and *Acinetobacter* spp. strains was evaluated against a panel of 22 and 16 antibiotics, respectively, commonly used in medical and veterinary practices (Oxoid, Basingstoke, UK) (Table S11). The quality control strain *E. coli* ATCC 25922 was used for verification purposes. Strains were categorized as multidrug-resistant (MDR) if they showed resistance to at least one antimicrobial agent from three or more categories. Strains resistant to at least one agent from all but two or fewer categories were classified as extensively drug-resistant (XDR), while those resistant to all agents were considered pan-drug-resistant (PDR) [67].

Additionally, the susceptibility of the strains to colistin was determined using the broth-microdilution method, following CLSI guidelines [66]. A stock solution of colistin sulfate (Sigma-Aldrich) was prepared, and concentrations ranging from 0.06 to 32 μg/mL were used. MICs of colistin were determined as the lowest concentration at which no visible growth was observed. *E. coli* ATCC 25922 served as the quality control strain.

### 4.4. Confirmation of β-Lactamase Production

In order to phenotypically characterize the *E. coli* and *K. pneumoniae* strains, the combination disk test was performed according to the protocols outlined by CLSI and EUCAST [66,68]. An inoculum was prepared as previously described and then spread onto Mueller Hinton agar plates. Antibiotic disks (Oxoid, Basingstoke, UK) containing ceftazidime (CAZ 30 μg), ceftazidime/clavulanic acid (CZC 30/10 μg), cefotaxime (CTX 30 μg), cefotaxime/clavulanic acid (CTC 30/10 μg), and cefoxitin (FOX 30 μg) were used. An increase of ≥ 5 mm in the zone diameter when the antibiotic was combined with clavulanic acid, compared to the zone diameter of the antibiotic alone, indicated an ESBL phenotype. Strains were classified as AmpC if they showed resistance to cefotaxime and ceftazidime without clavulanic acid induction and also exhibited resistance to cefoxitin. Strains were characterized as having both ESBL and AmpC phenotypes if they demonstrated an increase of ≥ 5 mm in the zone diameter with the combination antibiotic and clavulanic acid, compared to the antibiotic alone, along with resistance to cefoxitin. *E. coli* ATCC 25922 and *K. pneumoniae* ATCC 700603 were used as quality control strains.

### 4.5. Molecular Screening of β-Lactamase Genes

All β-lactam-resistant *Acinetobacter* strains, as well as *E. coli* and *K. pneumoniae* strains confirmed to exhibit an ESBL or AmpC phenotype or resistance to a carbapenem, underwent further molecular characterization to detect the presence of β-lactamase-producing genes. DNA extraction from pure cultures followed the method described by Peratikos et al. [65]. DNA quality and recovery were assessed using a NanoDrop microvolume spectrophotometer (Nanodrop 2000, Thermo Fisher Scientific, Waltham, MA, USA). Subsequently, DNA samples underwent four multiplex PCR and one simplex PCR to screen for common ESBL and AmpC-producing genes, following the protocol of Dallenne et al. [69], with modifications. The reaction mixture, prepared in a final volume of 25 μL, comprised 0.625U OneTaq™ DNA Polymerase (M0273S, NEB), 2.5 μL 10× OneTaq Standard Reaction Buffer (B9014S, NEB), 200 μM of dNTPs (N0447S, NEB), 0.2–0.5 μM of primers, and 2 μL of DNA sample (Table S12). *K. pneumoniae* ATCC 700603 served as the positive control. PCR was conducted in a thermal cycler (LabCycler Gradient, SensoQuest GmbH, Göttingen, Germany). Strains exhibiting resistance to at least one carbapenem were subjected to three additional multiplex PCR reactions to identify the presence of a carbapenemase gene, following the protocol outlined by Poirel et al. [53], whereas carbapenem-resistant *Acinetobacter* strains were also subjected to another multiplex PCR reaction to identify OXA-carbapenemases, as proposed by Woodford et al. [51]. In all PCR reactions for the detection of carbapenemase genes, the reaction mixture, prepared in a final volume of 25 μL, consisted of 0.625U OneTaq™ DNA Polymerase (M0273S, NEB), 2.5 μL 10× OneTaq Standard Reaction Buffer (B9014S, NEB), 200 μM of dNTPs (N0447S, NEB), 0.2 μM of primers, and 2 μL of DNA sample (Table S12). *K. pneumoniae* ATCC BAA-1705 was utilized as a positive control. The PCR products were visualized after electrophoresis on 1.5% agarose gels containing ethidium bromide using a UVP DigiDoc-It^®^ 125 gel imaging system (UVP, Cambridge, UK).

### 4.6. Phylogenetic Analysis of E. coli Strains

The PCR protocols outlined by Clermont et al. [70] were adapted to identify the phylogenetic groups of *E. coli* strains. The reaction mixture had a final volume of 25 μL and included 0.625U OneTaq™ DNA Polymerase (M0273S, NEB), 2.5 μL 10× OneTaq Standard Reaction Buffer (B9014S, NEB), 200 μM dNTPs (N0447S, NEB), 0.2–0.4 μM of primers, and 2 μL of DNA sample (Tables S13 and S14). *E. coli* ATCC 25922 was used as the positive control. The PCR products were then analyzed by electrophoresis on 1.5% agarose gels containing ethidium bromide using a UVP DigiDoc-It^®^ 125 gel imaging system (UVP, Cambridge, UK).

### 4.7. Statistical Analysis

Data were statistically analyzed using IBM SPSS Statistics software (v.29.0., IBM Corporation, Armonk, NY, USA). Measures of central tendency and dispersion were calculated to describe the data’s characteristics. Confidence intervals for prevalence were calculated using the Clopper–Pearson method (Binomial test). A Fisher’s exact test was employed to compare the prevalence of resistant strains in environmental and human samples across different seasons, as well as to compare questionnaire variables between humans testing positive and negative for resistant strains. A significance level of 5% (*p* ≤ 0.05) was used.

## 5. Conclusions

This study investigates the presence and characteristics of β-lactam-resistant *E. coli*, *K. pneumoniae*, and *Acinetobacter* spp. in hospital kitchens and among workers, using selective enrichment and isolation with ceftazidime and meropenem. To the best of our knowledge, this is the first study in Greece—where these AMR bacteria are endemic—focused on this issue. The findings show that hospital kitchens serve as reservoirs for multidrug-resistant strains, posing a significant public health risk. Moreover, this is likely the first report of AIM, DIM, and BIC carbapenemases in *Acinetobacter* strains from Greek hospitals. Concerning the contamination routes unveiled, almost all *E. coli* strains had a direct connection to chicken, *K. pneumoniae* seem to circulate between the kitchen and the clinics, and the *Acinetobacter* spp. circulation was undecisive since they occurred in a wide range of samples. The genetic relatedness of the strains underscores the persistent nature of them in the kitchen and clinical settings. The small distance levels observed between clinical and environmental strains suggests that hospital kitchens may play a role in the circulation of resistant strains within the hospital environment. Moreover, the results suggest that poultry can be a vehicle for the import of resistant strains from the outside environment to the hospital kitchen. The personnel did not seem to influence since only one person was found positive to the AMR bacteria of the study, perhaps due to the methodological approach followed. Therefore, the main points of entry of AMR bacteria to the kitchen seem to be the poultry and the returning food and equipment from the clinics. Further studies are needed to assess the presence of multidrug-resistant *E. coli*, *K. pneumoniae*, and *Acinetobacter* spp. within the hospital kitchens and verify the contamination routes to these premises. Expanding the study to include more hospitals, additional ESKAPE species, and a broader range of AMR genes would provide valuable insights, whereas employing molecular epidemiology techniques could also enhance strain characterization and map the transmission routes of AMR strains from hospital kitchens to wards and patients more effectively.

## Data Availability

Data are contained within the article or Supplementary Material. Further data can be available on request from the authors.

## Supplementary Materials

The following supporting information can be downloaded at: https://www.mdpi.com/article/10.3390/antibiotics13100934/s1, Table S1: Characterization of the resistant *Escherichia coli* strains; Table S2. Characterization of the resistant *Klebsiella pneumoniae* strains; Table S3. Characterization of the resistant *Acinetobacter* strains; Table S4. Demographic characteristics of kitchen staff; Table S5. Characteristics and behaviors of hospital staff regarding antibiotic use; Table S6. State of health of kitchen staff; Table S7. Antibiotic use in kitchen staff; Table S8. Description of the collected samples; Table S9. List of questions, regarding medical history and history of antibiotic consumption in humans; Table S10. Description of clinical strains used in the study; Table S11. Antibiotic disks used for antibiotic susceptibility with the disk diffusion method; Table S12. Primers used for molecular screening of β-lactamase genes; Table S13. Primers used for phylogenetic analysis of *E. coli* strains; Table S14. Quadruplex PCR results and categorization of *E. coli* strains to phylogenetic groups.
