# Supplementary material for: Prevalence, Characterization, and Epidemiological Relationships between ESBL and Carbapenemase-Producing Escherichia coli, Klebsiella pneumoniae, and Acinetobacter spp. Isolated from Humans and the Kitchen Environment of Two Greek Hospitals
Source: Antibiotics (Basel). 2024 Oct 2;13(10):934. doi: 10.3390/antibiotics13100934 (PMC11504295; doi:10.3390/antibiotics13100934)
Supplement: Supplementary file 1 [file antibiotics-13-00934-s001.zip › antibiotics-3209774-supplementary.pdf]

Table S1. Characterization of the resistant *Escherichia coli* strains.

Strain I.D.	Sample	Region	Season	Resistance profile	MIC Colistin (μg/mL)	Characterization of resistance profile	β-lactam resistance profile	β – lactamase genes	Phylogenetic group
E1	Sink (chicken)	Epirus	Spring	AM, AMC, AZM, CAZ, CIP, CN, CTX, DO, FEP, SXT, TE, TOB, TPZ	0.06	MDR	ESBL	CTX-M group 1	A
E2	Sink (chicken)	Epirus	Summer	AM, AMC, AZM, CAZ, CHL, CIP, CTX, FEP, LEV, SXT, TIM	0.125	MDR	ESBL	TEM, CTX-M group 1	A
E3	Chicken	Epirus	Summer	AM, AZM, CAZ, CTX, DO, FEP, SXT, TE	0.06	MDR	ESBL	CTX-M group 1	A
E4	Surface (chicken cutting board)	Central Macedonia	Summer	AM, AMC, CAZ, CTX, TE	0.06	MDR	ESBL	-	A
E5	Surface (surface around equipment washing up)	Central Macedonia	Summer	AM, AMC, CAZ, CTX, TE	0.06	MDR	ESBL	SHV	Unknown
E6	Sink (chicken)	Central Macedonia	Summer	AM, CAZ, CTX, TE	0.06	MDR	ESBL	-	A
E7	Equipment (chicken knife)	Central Macedonia	Summer	AM, CAZ, CTX, TE, TPZ	0.06	MDR	ESBL	SHV	A
E8	Equipment (sponge towel – chicken)	Central Macedonia	Summer	AM, CAZ, CTX, TE	0.06	MDR	ESBL	SHV	A
E9	Chicken	Central Macedonia	Summer	AM, AMC, AZM, CAZ, CTX, DO, FEP, SXT, TE, TPZ	0.06	MDR	ESBL	SHV	A
E10	Chicken	Central Macedonia	Summer	AM, AMC, AZM, CAZ, CTX, DO, SAM, TE, TPZ	0.06	MDR	ESBL	SHV	A
E11	Chicken	Central Macedonia	Summer	AM, AMC, CAZ, CTX, FEP, TE, TPZ	0.06	MDR	ESBL	SHV	A
E12	Chicken	Central Macedonia	Summer	AM, AMC, AZM, CAZ, CIP, CTX, DO, FEP, SXT, TE, TOB, TPZ	0.06	MDR	ESBL	-	Unknown
E13	Chicken	Central Macedonia	Summer	AM, AMC, CAZ, CHL, CIP, CTX, DO, FEP, LEV, TE, TPZ	0.06	MDR	ESBL	CTX-M group 1	B1
E14	Chicken	Epirus	Autumn	AK, AM, AZM, CAZ, CIP, CTX, DO, FEP, SXT, TE, TOB	0.06	MDR	ESBL	-	A
E15	Chicken	Epirus	Autumn	AM, CAZ, CIP, CTX, DO, FEP, SXT, TE, TPZ	0.05	MDR	ESBL	TEM, CTX-M group 1	A
E16	Chicken	Central Macedonia	Winter	AK, AM, CAZ, CIP, CN, CTX, TE, TPZ	0.06	MDR	ESBL	SHV	A

Strain I.D.	Sample	Region	Season	Resistance profile	MIC Colistin (μg/mL)	Characterization of resistance profile	β-lactam resistance profile	β – lactamase genes	Phylogenetic group
E17	Sink (chicken)	Epirus	Winter	AM, AZM, CAZ, CIP, CN, CTX, DO, FEP, SXT, TE, TPZ	0.06	MDR	ESBL	CTX-M group 1	A
E18	Surface (vegetable cutting board)	Central Macedonia	Spring	AK, AM, AMC, AZM, CAZ, CHL, CIP, CTX, DO, FEP, LEV, SXT, TE, TPZ	0.125	MDR	ESBL	CTX-M group 1	B1
E19	Chicken	Central Macedonia	Spring	AK, AM, AMC, AZM, CAZ, CIP, CN, CTX, FEP, TE, TOB, TPZ	0.125	MDR	ESBL	SHV	A
E20	Chicken	Central Macedonia	Spring	AK, AM, AMC, AZM, CAZ, CHL, CIP, CN, CTX, DO, FEP, LEV, SAM, SXT, TE, TIM, TOB, TPZ	0.125	MDR	ESBL	TEM, CTX-M group 1	A
E21	Chicken	Central Macedonia	Spring	AK, AM, AMC, AZM, CAZ, CHL, CIP, CN, CTX, DO, FEP, LEV, SAM, SXT, TE, TIM, TPZ	0.25	MDR	ESBL	TEM, CTX-M group 1	B1
E22	Chicken	Central Macedonia	Spring	AM, AMC, AZM, CAZ, CHL, CIP, CN, CTX, DO, FEP, LEV, SXT, TE, TIM, TOB, TPZ	0.125	MDR	ESBL	CTX-M group 1	B1
E23	Chicken	Central Macedonia	Spring	AM, CAZ, CIP, CN, CTX, FEP, LEV, SXT, TE, TPZ	0.125	MDR	ESBL	SHV	A

Table S2. Characterization of the resistant *Klebsiella pneumoniae* strains.

Strain I.D.	Sample	Region	Season	Resistance profile	MIC Colistin (μg/mL)	Characterization of resistance profile	β-lactam resistance profile	β – lactamase genes
K1	Utensils used by hospital patients	Epirus	Spring	AK, AM, AMC, AZM, CAZ, CHL, CIP, CTX, ETP, FEP, FOX, IPM, LEV, MEM, SAM, SXT, TIM, TOB, TPZ	0.25	MDR	Carbapenemases	TEM, SHV, KPC
K2	Utensils used by hospital patients	Epirus	Spring	AK, AM, AMC, AZM, CAZ, CHL, CIP, CTX, ETP, FEP, FOX, IPM, LEV, MEM, SAM, SXT, TIM, TOB, TPZ	1	MDR	Carbapenemases	TEM, SHV, KPC
K3	Utensils used by hospital patients	Epirus	Spring	AK, AM, AMC, AZM, CAZ, CHL, CIP, CN, CTX, ETP, FEP, FOX, IPM, LEV, MEM, SAM, SXT, TIM, TOB, TPZ	0.25	MDR	Carbapenemases	TEM, SHV, KPC
K4	Utensils used by hospital patients	Epirus	Spring	AK, AM, AMC, AZM, CAZ, CHL, CIP, CN, COL, CTX, ETP, FEP, FOX, IPM, LEV, MEM, SAM, SXT, TIM, TOB, TPZ	8	MDR	Carbapenemases	KPC
K5	Clinical isolate (urine, Internal Medicine)	Central Macedonia	Summer	AK, AM, AMC, AZM, CAZ, CIP, CTX, DO, ETP, FEP, FOX, IPM, LEV, MEM, SAM, SXT, TE, TIM, TPZ	0.06	MDR	Carbapenemases	TEM, SHV, CTX-M group 1, PER, VEB, NDM
K6	Clinical isolate (pharyngeal, ICU)	Central Macedonia	Summer	AM, AMC, AZM, CAZ, CHL, CIP, CTX, DO, ETP, FEP, FOX, IPM, LEV, MEM, SAM, SXT, TE, TIM, TPZ	0.5	MDR	Carbapenemases	TEM, SHV, CTX-M group 1, PER, VEB, NDM
K7	Clinical isolate (pharyngeal, ICU)	Central Macedonia	Summer	AM, AMC, AZM, CAZ, CHL, CIP, CTX, DO, ETP, FEP, FOX, IPM, LEV, MEM, SAM, SXT, TE, TIM, TPZ	0.25	MDR	Carbapenemases	TEM, SHV, CTX-M group 1, PER, VEB, NDM
K8	Clinical isolate (pharyngeal, ICU)	Central Macedonia	Summer	AK, AM, AMC, AZM, CAZ, CIP, CN, CTX, DO, ETP, FEP, FOX, IPM, LEV, MEM, SAM, SXT, TE, TIM, TOB, TPZ	0.06	MDR	Carbapenemases	OXA-1, CTX-M group 1
K9	Clinical isolate (underarm, ICU))	Central Macedonia	Autumn	AK, AM, AMC, AZM, CAZ, CIP, CN, CTX, DO, ETP, FEP, FOX, IPM, LEV, MEM, SAM, SXT, TE, TIM, TOB, TPZ	0.06	MDR	Carbapenemases	TEM, SHV, CTX-M group 1, PER, VEB, NDM, OXA-48-like
K10	Clinical isolate (pharyngeal, ICU)	Central Macedonia	Autumn	AK, AM, AMC, AZM, CAZ, CIP, CN, CTX, DO, ETP, FEP, FOX, LEV, MEM, SAM, SXT, TE, TIM, TOB, TPZ	0.125	MDR	Carbapenemases	OXA-1, CTX-M group 1
K11	Clinical isolate (pharyngeal, ICU)	Central Macedonia	Autumn	AK, AM, AMC, AZM, CAZ, CIP, CN, CTX, DO, ETP, FEP, FOX, IPM, LEV, MEM, SAM, SXT, TE, TIM, TOB, TPZ	0.06	MDR	Carbapenemases	SHV, OXA-1, CTX-M group 1, NDM
K12	Clinical isolate (bronchial, ICU)	Central Macedonia	Autumn	AK, AM, AMC, AZM, CAZ, CIP, CN, CTX, DO, ETP, FEP, FOX, IPM, LEV, MEM, SAM, SXT, TE, TIM, TOB, TPZ	0.06	MDR	Carbapenemases	TEM, SHV, CTX-M group 1, PER, VEB, NDM, OXA-48-like
K13	Clinical isolate (bronchial, ICU)	Central Macedonia	Autumn	AK, AM, AMC, AZM, CAZ, CIP, CN, CTX, DO, ETP, FEP, FOX, IPM, LEV, MEM, SAM, SXT, TE, TIM, TOB, TPZ	0.06	MDR	Carbapenemases	TEM, SHV, CTX-M group 1, PER, VEB, NDM, OXA-48-like

Strain I.D.	Sample	Region	Season	Resistance profile	MIC Colistin (μg/mL)	Characterization of resistance profile	β-lactam resistance profile	β – lactamase genes
K14	Clinical isolate (underarm, ICU))	Central Macedonia	Autumn	AK, AM, AMC, AZM, CAZ, CIP, CN, CTX, DO, ETP, FEP, FOX, LEV, MEM, SAM, SXT, TE, TIM, TOB, TPZ	0.06	MDR	Carbapenemases	SHV, OXA-1, CTX-M group 1, NDM, OXA-48-like
K15	Clinical isolate (pharyngeal, Internal Medicine)	Central Macedonia	Autumn	AK, AM, AMC, AZM, CAZ, CIP, CN, COL, CTX, ETP, FEP, FOX, IPM, LEV, MEM, SAM, SXT, TIM, TOB, TPZ	8	MDR	Carbapenemases	SHV, KPC
K16	Clinical isolate (pharyngeal, Internal Medicine)	Central Macedonia	Autumn	AK, AM, AMC, AZM, CAZ, CIP, CTX, ETP, FEP, FOX, IPM, LEV, MEM, SAM, SXT, TIM, TOB, TPZ	1	MDR	Carbapenemases	SHV, KPC
K17	Clinical isolate (nasal, ICU)	Central Macedonia	Winter	AK, AM, AMC, AZM, CAZ, CHL, CIP, CN, CTX, DO, ETP, FEP, FOX, IPM, LEV, MEM, SAM, SXT, TE, TIM, TOB, TPZ	0.125	MDR	Carbapenemases	TEM, SHV, CTX-M group 1, VEB, NDM, OXA-48-like
K18	Clinical isolate (pharyngeal, ICU)	Central Macedonia	Winter	AK, AM, AMC, AZM, CAZ, CHL, CIP, CN, CTX, DO, ETP, FEP, FOX, IPM, LEV, MEM, SAM, SXT, TE, TIM, TOB, TPZ	0.5	MDR	Carbapenemases	TEM, SHV, CTX-M group 1, VEB, NDM, OXA-48-like
K19	Clinical isolate (pharyngeal, ICU)	Central Macedonia	Winter	AK, AM, AMC, AZM, CAZ, CIP, CN, CTX, DO, ETP, FEP, FOX, IPM, LEV, MEM, SAM, SXT, TE, TIM, TOB, TPZ	0.06	MDR	Carbapenemases	SHV, OXA-1, CTX-M group 1, NDM
K20	Clinical isolate (pharyngeal, ICU)	Central Macedonia	Winter	AK, AM, AMC, AZM, CAZ, CIP, CN, CTX, ETP, FEP, FOX, IPM, LEV, MEM, SAM, SXT, TIM, TOB, TPZ	0.125	MDR	Carbapenemases	SHV, OXA-1, CTX-M group 1, NDM
K21	Utensils used by hospital patients	Central Macedonia	Spring	AK, AM, AMC, AZM, CAZ, CIP, CN, CTX, DO, ETP, FEP, FOX, IPM, LEV, MEM, SAM, TE, TIM, TOB, TPZ	0.125	MDR	Carbapenemases	SHV, CTX-M group 1, KPC
K22	Clinical isolate (bronchial, ICU)	Central Macedonia	Spring	AK, AM, AMC, AZM, CAZ, CIP, CN, COL, CTX, DO, ETP, FEP, FOX, IPM, LEV, MEM, SAM, SXT, TE, TIM, TOB, TPZ	32	MDR	Carbapenemases	TEM, SHV, OXA-1, CTX-M group 1, VEB, KPC
K23	Clinical isolate (bronchial, ICU)	Central Macedonia	Spring	AK, AM, AMC, AZM, CAZ, CHL, CIP, CN, CTX, DO, ETP, FEP, FOX, IPM, LEV, MEM, SAM, SXT, TE, TIM, TOB, TPZ	0.5	MDR	Carbapenemases	TEM, SHV, CTX-M group 1, VEB, KPC, NDM
K24	Clinical isolate (bronchial, ICU)	Central Macedonia	Spring	AK, AM, AMC, AZM, CAZ, CHL, CIP, CN, CTX, DO, ETP, FEP, FOX, IPM, LEV, MEM, SAM, SXT, TE, TIM, TOB, TPZ	0.5	MDR	Carbapenemases	TEM, SHV, CTX-M group 1, VEB, KPC, NDM

Table S3. Characterization of the resistant *Acinetobacter* strains.

Strain I.D.	Isolate	Sample	Region	Season	Resistance profile	MIC Colistin (μg/mL)	Characterization of resistance profile	β – lactamase genes
A1	*A. baumannii*	Surface (Surface around equipment washing-up)	Epirus	Spring	CAZ, CTX, PRL, TPZ	0.25	MDR	-
A2	*A. baumannii*	Serving Tray	Epirus	Spring	CAZ, CTX, PRL, SXT, TPZ	0.125	MDR	-
A3	*A. baumannii*	Serving Tray	Epirus	Spring	CAZ, CTX, PRL, TPZ	0.06	MDR	-
A4	*A. baumannii*	Sink (cheese)	Epirus	Summer	CAZ, CTX, PRL, TPZ	0.125	MDR	-
A5	*A. baumannii*	Sink (cheese)	Epirus	Summer	AK, CAZ, CIP, CN, CTX, DO, FEP, IPM, LEV, MEM, PRL, SAM, SXT, TIM, TOB, TPZ	0.06	XDR	OXA-51
A6	*A. baumannii*	Sink (washing up)	Epirus	Summer	CAZ, CTX, PRL, TPZ	0.125	MDR	-
A7	*A. calcoaceticus*	Sink (washing up)	Epirus	Summer	CAZ, CTX, PRL, TPZ	0.125	MDR	-
A8	*A. baumannii*	Utensils used by hospital patients	Epirus	Summer	AK, CAZ, CIP, CN, CTX, DO, FEP, IPM, LEV, MEM, PRL, SAM, SXT, TIM, TOB, TPZ	0.25	XDR	TEM, NDM, OXA-48, AIM, OXA-23, OXA-51
A9	*A. calcoaceticus*	Equipment (vegetable knife)	Epirus	Summer	AK, CAZ, CIP, CN, CTX, DO, FEP, IPM, LEV, MEM, PRL, SAM, SXT, TIM, TOB, TPZ	0.125	XDR	BIC, OXA-51
A10	*A. pittii*	Sink (vegetables)	Epirus	Summer	AK, CAZ, CIP, CN, CTX, DO, FEP, IPM, LEV, MEM, PRL, SAM, SXT, TIM, TOB, TPZ	0.125	XDR	SIM
A11	*A. pittii*	Utensils used by hospital patients	Epirus	Summer	CAZ, CTX, PRL, SAM, SXT, TIM, TPZ	0.125	MDR	-
A12	*A. baumannii*	Surface (Surface of food preparation)	Central Macedonia	Summer	CAZ, CTX, PRL	0.25	-	-
A13	*A. pittii*	Sponge (food preparation)	Central Macedonia	Summer	CAZ, CTX, PRL, TPZ	0.25	MDR	-
A14	*A. baumannii*	Sponge towel (food preparation)	Central Macedonia	Summer	CAZ, CTX, PRL	0.25	-	TEM
A15	*A. baumannii*	Utensils used by hospital patients	Central Macedonia	Summer	AK, CAZ, CIP, CN, CTX, DO, FEP, IPM, LEV, MEM, PRL, SAM, SXT, TIM, TOB, TPZ	0.125	XDR	CTX-M group 1, CTX-M group 9, AIM, OXA-23, OXA-51
A16	*A. baumannii*	Utensils used by hospital patients	Central Macedonia	Summer	AK, CAZ, CIP, CN, CTX, DO, FEP, IPM, LEV, MEM, PRL, SXT, TIM, TOB, TPZ	0.125	XDR	CTX-M group 1, CTX-M group 9, AIM, DIM, OXA-23, OXA-51

Strain I.D.	Isolate	Sample	Region	Season	Resistance profile	MIC Colistin (μg/mL)	Characterization of resistance profile	β – lactamase genes
A17	*A. baumannii*	Serving Tray	Central Macedonia	Summer	AK, CAZ, CIP, CN, CTX, DO, FEP, IPM, LEV, MEM, PRL, SAM, SXT, TIM, TOB, TPZ	0.25	XDR	CTX-M group 1, VIM, AIM, DIM, OXA-23, OXA-51
A18	*A. baumannii*	Table	Central Macedonia	Summer	CAZ, CTX, PRL, SXT, TPZ	0.06	MDR	-
A19	*A. baumannii*	Clinical isolate (nasal, orthopedics)	Central Macedonia	Summer	AK, CAZ, CIP, CN, CTX, DO, FEP, IPM, LEV, MEM, PRL, SAM, SXT, TIM, TOB, TPZ	0.125	XDR	OXA-23, OXA-51
A20	*A. baumannii*	Clinical isolate (nasal, orthopedics)	Central Macedonia	Summer	AK, CAZ, CIP, CN, CTX, DO, FEP, IPM, LEV, MEM, PRL, SAM, SXT, TIM, TOB, TPZ	0.125	XDR	OXA-23, OXA-51
A21	*A. pittii*	Human skin (chef)	Central Macedonia	Summer	CAZ, CTX, PRL, TPZ	0.125	MDR	-
A22	*A. baumannii*	Human skin (chef)	Central Macedonia	Summer	CAZ, CTX, FEP, PRL, TPZ	0.125	MDR	-
A23	*A. nosocomialis*	Sink (food preparation)	Central Macedonia	Summer	CAZ, CTX, PRL, TPZ	0.25	MDR	-
A24	*A. nosocomialis*	Sponge (food preparation)	Central Macedonia	Summer	CAZ, CTX, PRL	0.125	-	-
A25	*A. baumannii*	Clinical isolate (pharyngeal, ICU)	Central Macedonia	Summer	AK, CAZ, CIP, CN, CTX, DO, FEP, IPM, LEV, MEM, PRL, SAM, SXT, TIM, TOB, TPZ	1	XDR	CTX-M group 1, CTX-M group 9, VIM, AIM, DIM, OXA-23, OXA-51
A26	*A. pittii*	Utensils used by hospital patients	Epirus	Autumn	CAZ, CTX, PRL, SXT, TPZ	0.25	MDR	-
A27	*A. baumannii*	Serving Tray	Epirus	Autumn	CAZ, CTX, PRL	0.25	-	-
A28	*A. baumannii*	Clinical isolate (nasal, ICU)	Central Macedonia	Autumn	AK, CAZ, CIP, CN, COL, CTX, DO, FEP, IPM, LEV, MEM, PRL, SAM, SXT, TIM, TOB, TPZ	8	PDR	OXA-23, OXA-51
A29	*A. baumannii*	Clinical isolate (nasal, ICU)	Central Macedonia	Autumn	AK, CAZ, CIP, CN, COL, CTX, DO, FEP, IPM, LEV, MEM, PRL, SAM, SXT, TIM, TOB, TPZ	4	PDR	VIM, AIM, DIM, OXA-23, OXA-51
A30	*A. baumannii*	Surface (meat cutting board)	Central Macedonia	Autumn	CAZ, CTX, PRL, TPZ	0.25	-	-
A31	*A. baumannii*	Sink (food preparation)	Central Macedonia	Autumn	CAZ, CTX, PRL, SXT, TPZ	1	MDR	-

Strain I.D.	Isolate	Sample	Region	Season	Resistance profile	MIC Colistin (μg/mL)	Characterization of resistance profile	β – lactamase genes
A32	*A. baumannii*	Utensils used by hospital patients	Central Macedonia	Autumn	CAZ, CTX, PRL, TPZ	0.125	MDR	-
A33	*A. baumannii*	Table	Central Macedonia	Autumn	CAZ, CTX, PRL, SXT, TPZ	0.06	MDR	-
A34	*A. baumannii*	Clinical isolate (underarm, ICU))	Central Macedonia	Autumn	AK, CAZ, CIP, CN, CTX, DO, FEP, IPM, LEV, MEM, PRL, SAM, SXT, TIM, TOB, TPZ	0.125	XDR	OXA-23, OXA-51
A35	*A. baumannii*	Clinical isolate (pharyngeal, ICU)	Central Macedonia	Autumn	AK, CAZ, CIP, CN, CTX, DO, FEP, IPM, LEV, MEM, PRL, SAM, SXT, TIM, TOB, TPZ	0.25	XDR	VIM, OXA-23, OXA-51
A36	*A. baumannii*	Clinical isolate (pharyngeal, ICU)	Central Macedonia	Autumn	AK, CAZ, CIP, CN, CTX, DO, FEP, IPM, LEV, MEM, PRL, SAM, SXT, TIM, TOB, TPZ	0.125	XDR	VIM, OXA-23, OXA-51
A37	*A. baumannii*	Clinical isolate (underarm, ICU))	Central Macedonia	Autumn	AK, CAZ, CIP, CN, CTX, DO, FEP, IPM, LEV, MEM, PRL, SAM, SXT, TIM, TOB, TPZ	0.125	XDR	VIM, OXA-23, OXA-51
A38	*A. calcoaceticus*	Equipment (food preparation utensils)	Central Macedonia	Autumn	CAZ, CTX, PRL, TPZ	0.25	MDR	-
A39	*A. baumannii*	Sink (vegetables)	Central Macedonia	Autumn	CAZ, CTX, PRL, TPZ	0.25	MDR	CTX-M group 1, CTX-M-2
A40	*A. calcoaceticus*	Surface (Surface of food preparation)	Central Macedonia	Autumn	CAZ, CTX, PRL, TPZ	0.06	MDR	-
A41	*A. baumannii*	Surface (cheese cutting board)	Central Macedonia	Winter	CAZ, CTX, PRL	0.5	-	-
A42	*A. baumannii*	Equipment (sponge towel – cheese)	Central Macedonia	Winter	CAZ, CTX, PRL, TPZ	0.125	MDR	-
A43	*A. baumannii*	Sink (kitchen staff)	Central Macedonia	Winter	CAZ, CTX, PRL, TPZ	0.25	MDR	-
A44	*A. baumannii*	Sink (food preparation)	Central Macedonia	Winter	CAZ, CTX, PRL, SXT, TPZ	0.5	MDR	-
A45	*A. baumannii*	Clinical isolate (underarm, ICU))	Central Macedonia	Winter	AK, CAZ, CIP, CN, CTX, DO, FEP, IPM, LEV, MEM, PRL, SAM, SXT, TIM, TOB, TPZ	0.5	XDR	TEM, VIM, OXA-23, OXA-51
A46	*A. baumannii*	Clinical isolate (nasal, ICU)	Central Macedonia	Winter	AK, CAZ, CIP, CN, COL, CTX, DO, FEP, IPM, LEV, MEM, PRL, SAM, SXT, TIM, TOB, TPZ	8	PDR	OXA-23, OXA-51

Strain I.D.	Isolate	Sample	Region	Season	Resistance profile	MIC Colistin (μg/mL)	Characterization of resistance profile	β – lactamase genes
A47	*A. baumannii*	Clinical isolate (pharyngeal, ICU)	Central Macedonia	Winter	AK, CAZ, CIP, CN, COL, CTX, DO, FEP, IPM, LEV, MEM, PRL, SAM, SXT, TIM, TOB, TPZ	4	PDR	OXA-23, OXA-51
A48	*A. baumannii*	Clinical isolate (pharyngeal, ICU)	Central Macedonia	Winter	AK, CAZ, CIP, CN, COL, CTX, DO, FEP, IPM, LEV, MEM, PRL, SAM, SXT, TIM, TOB, TPZ	8	PDR	OXA-23, OXA-51
A49	*A. baumannii*	Clinical isolate (pharyngeal, ICU)	Central Macedonia	Winter	AK, CAZ, CIP, CN, COL, CTX, DO, FEP, IPM, LEV, MEM, PRL, SAM, SXT, TIM, TOB, TPZ	4	PDR	TEM, OXA-23, OXA-51
A50	*A. baumannii*	Clinical isolate (pharyngeal, ICU)	Central Macedonia	Winter	AK, CAZ, CIP, CN, CTX, DO, FEP, IPM, LEV, MEM, PRL, SAM, SXT, TIM, TOB, TPZ	2	XDR	TEM, OXA-23, OXA-51
A51	*A. pittii*	Equipment (vegetable knife)	Central Macedonia	Spring	CAZ, CIP, CTX, PRL, TPZ	0.25	MDR	-
A52	*A. pitti*	Equipment (meat knife)	Central Macedonia	Spring	CAZ, CTX, PRL, SXT, TPZ	0.25	MDR	-
A53	*A. baumannii*	Table	Central Macedonia	Spring	CAZ, CTX, PRL, SXT, TPZ	0.125	MDR	-
A54	*A. baumannii*	Clinical isolate (bronchial, ICU)	Central Macedonia	Spring	AK, CAZ, CIP, CN, COL, CTX, DO, FEP, IPM, LEV, MEM, PRL, SAM, SXT, TIM, TOB, TPZ	16	PDR	TEM, DIM, OXA-23, OXA-51

Table S4. Demographic characteristics of kitchen staff.

Human I.D.	Region	Seasons of sampling	Gender	Age	Occupation	Place of residence	Smoking (moment of speaking)	Pack years	Alcohol consumption (units/week)
H1	Central Macedonia	Summer, Autumn	Male	63	Chef	Giannitsa (Town)	No	0	17.5
H2	Central Macedonia	Winter, Spring	Female	57	Nurse	Giannitsa (Town)	Yes	15	2.5
H3	Central Macedonia	Autumn, Winter, Spring	Female	34	Chef	Pentaplatanos (Village)	Yes	15	0
H4	Central Macedonia	Autumn, Winter, Spring	Female	44	Chef	Pella (Village)	No	0	0
H5	Central Macedonia	Summer, Autumn, Winter, Spring	Female	24	Chef	Mylotopos (Village)	No	0	0.6
H6	Central Macedonia	Summer	Female	44	Chef	Giannitsa (Town)	No	0	2.5
H7	Central Macedonia	Summer	Female	47	Food service worker	Giannitsa (Town)	Yes	30	0
H8	Central Macedonia	Summer	Female	48	Cleaning staff	Laka (Village)	Yes	30	2.5
H9	Central Macedonia	Autumn	Male	24	Chef	Giannitsa (Town)	Yes	6	0
H10	Central Macedonia	Winter	Female	59	Cleaning staff	Kria Vrisi (Village)	No	30	0
H11	Central Macedonia	Spring	Female	43	Nurse	Giannitsa (Town)	No	0	2.5
H12	Epirus	Spring, Summer, Winter	Female	44	Food service worker	Ioannina (City)	No	25	0
H13	Epirus	Summer, Autumn, Winter	Female	37	Chef	Ioannina (City)	No	0	12.5
H14	Epirus	Spring, Summer, Winter	Female	41	Chef	Platania (Village)	No	0	0
H15	Epirus	Spring	Female	54	Cleaning staff	Anatoli (Village)	Yes	15	0
H16	Epirus	Spring	Female	41	Food service worker	Ioannina (City)	No	2,5	0
H17	Epirus	Spring	Female	30	Food service worker	Ioannina (City)	No	0	2.5
H18	Epirus	Summer	Female	36	Food service worker	Pedini (Village)	No	0	2.5

Human I.D.	Region	Seasons of sampling	Gender	Age	Occupation	Place of residence	Smoking (moment of speaking)	Pack years	Alcohol consumption (units/week)
H19	Epirus	Summer	Female	34	Chef	Eleousa (Village)	Yes	6	2.5
H20	Epirus	Autumn	Female	37	Food service worker	Ioannina (City)	Yes	7,5	0.6
H21	Epirus	Autumn	Female	36	Food service worker	Perama (Village)	Yes	10	15
H22	Epirus	Autumn	Female	42	Food service worker	Ioannina (City)	No	17	1.25
H23	Epirus	Autumn	Female	52	Cleaning staff	Ioannina (City)	No	0	0.6
H24	Epirus	Winter	Female	49	Chef	Kardamitsa (Village)	Yes	15	2.5
H25	Epirus	Winter	Female	31	Food service worker	Kardamitsa (Village)	No	0	2.5

Table S5. Characteristics and behaviors of hospital staff regarding antibiotic use.

Variable	Total Number (%)	p-value
**Gender**		
Male	2 (8.0%)	0.075
Female	23 (92.0%)	
**Age**		
≤40	10 (40.0%)	1.0
41-64	15 (60.0%)	
≥65	0 (0.0%)	
**Smoking** (moment of speaking)		
Yes	10 (40.0%)	1.0
No	15 (60.0%)	

**Variable**	**Total Number (%)**	**p-value**
**Pack years**		
0	11 (44.0%)	
1-19	10 (40.0%)	1.0
20-39	4 (16.0%)	
≥40	0 (0.0%)	
**Alcohol consumption** (units/week)		
0	9 (26.0%)	
0.1-2.5	13 (52.0%)	0.15
2.6-10	0 (0.0%)	
≥10.1	3 (12.0%)	
**Current health disorders / comorbidities**		
Yes	13 (52.0%)	0.475
No	12 (48.0%)	
**Immunosuppression**		
Yes	2 (8.0%)	1.0
No	23 (92.0%)	
**Recent vaccinations** (e.g. Covid-19, Influenza, Pneumococcus etc.)		1.0
Yes	23 (92.0%)	
No	2 (8.0%)	
**Concomitant therapies**		0.45

Variable	Total Number (%)	p-value
Yes	12 (48.0%)	
No	13 (52.0%)	
**Family history**		
Yes	11 (44.0%)	0.475
No	14 (56.0%)	
**Recent surgical procedures**		
Yes	2 (8.0%)	1.0
No	23 (92.0%)	
**Allergies**		
Yes	9 (36.0%)	0.35
No	16 (64.0%)	
**Previous hospitalization**		
Yes	2 (8.0%)	1.0
No	23 (92.0%)	
**Recent antibiotic consumption**		
**Spring**		
Yes	4 (40.0%)	
No	6 (60.0%)	1.0
**Summer**		
Yes	1 (10.0%)	
No	9 (90.0%)	

Variable	Total Number (%)	p-value
**Autumn**		
Yes	4 (40.0%)	
No	6 (60.0%)	
**Winter**		
Yes	6 (60.0%)	
No	4 (40.0%)	
**Reasons for antibiotic consumption**		
Treatment	25 (100.0%)	1.0
Prophylaxis	3 (12.0%)	
Metaphylaxis	0 (0.0%)	
**Stock of antibiotics in house**		
Yes	7 (28.0%)	0.25
No	18 (72.0%)	
**Use of antibiotics before their expiration**		
Yes	25 (100.0%)	-
No	0 (0.0%)	
**Frequency and easiness of antibiotic consumption**		
Never (only when absolutely necessary)	20 (80.0%)	1.0
Rare	2 (8.0%)	

Variable	Total Number (%)	p-value
Sometimes	2 (8.0%)	
Very often	1 (4.0%)	
**Antibiotic consumption after doctor's prescription**		
Always	22 (88.0%)	1.0
Usually	3 (12.0%)	
Sometimes	0 (0.0%)	
Rarely	0 (0.0%)	
**Microbial culture test and antibiogram performed**		
Always	0 (0.0%)	1.0
Usually	0 (0.0%)	
Sometimes	1 (4.0%)	
Never	24 (96.0%)	
**Guidance by doctor**		
Complete	0 (0.0%)	-
Partial	25 (100.0%)	
None	0 (0.0%)	

Table S6. State of health of kitchen staff.

Human I.D.	Region	Current health disorders / comorbidities	Immunosuppression	Vaccination	Concomitant therapies	Family history	Surgical procedures	Allergies	Previous hospitalization
H1	Central Macedonia	Asthma, Nasal turbinate hypertrophy	No	Covid-19, Influenza, Pneumococcus	Antihistamine spray	Coronary heart disease, diabetes mellitus	No	Brazilian nut	No
H2	Central Macedonia	Hypothyroidism	No	Covid-19, Influenza	Levothyroxine	Colon cancer, diabetes mellitus	No	No	No
H3	Central Macedonia	No	No	Covid-19	No	No	No	No	No
H4	Central Macedonia	Bell's Palsy, Dyslipidemia	No	Covid-19, Pneumococcus	Vitamin D, calcium, levothyroxine, thiamine, rosuvastatin	Atrial fibrillation	Thyroidectomy	No	No
H5	Central Macedonia	No	No	Covid-19	No	No	No	No	No
H6	Central Macedonia	Iron deficiency anaemia	No	Covid-19	Vitamin B12, ferum, folic acid	Atrial fibrillation	No	Eczema	No
H7	Central Macedonia	Dyslipidemia	No	Covid-19	Atorvastatin	No	No	No	No
H8	Central Macedonia	Hashimoto, Atopic dermatitis	No	Covid-19	Vitamin D, levothyroxine	No	Endometrial ablation (in 2023)	No	No
H9	Central Macedonia	No	No	Covid-19	No	No	No	No	No
H10	Central Macedonia	Arterial hypertension, allergic rhinitis	No	No	Nebivolol, levocetirizine	Myocardial infarction, pancreatic cancer,	No	Dust, grass, cereal, amoxicillin /	No

Human I.D.	Region	Current health disorders / comorbidities	Immunosuppression	Vaccination	Concomitant therapies	Family history	Surgical procedures	Allergies	Previous hospitalization
						diabetes mellitus		clavulanic acid	
H11	Central Macedonia	Hashimoto, Iron deficiency anaemia	No	Covid-19, Influenza	Fluoxetine, levothyroxine	Schizophrenia, artial fibrillation	No	No	No
H12	Epirus	No	No	Covid-19	No	Sarcoma, diabetes mellitus, coronary heart disease	No	Seasonal allergies, aspirin	No
H13	Epirus	No	No	Covid-19	No	No	No	No	No
H14	Epirus	Multiple sclerosis, anaemia	Natalizumab	Covid-19	Natalizumab	Dyslipidemia, arterial ischemic stroke	No	Paramagnetic elements	Yes
H15	Epirus	Hypothyroidism, Tachycardia, Arterial hypertension, Iron deficiency anaemia	No	Covid-19	Levothyroxine, bisoprorol, ferum supplement	No	No	No	No
H16	Epirus	Gastroesophageal reflux disease	No	No	Omeprazole	Arterial hypertension, diabetes mellitus	No	Amoxicillin, azithromycin, niflumic acid	No
H17	Epirus	No	No	Covid-19	No	Hypothyroidism	No	No	No
H18	Epirus	No	No	Covid-19	No	No	No	No	No
H19	Epirus	Albuminuria	No	Covid-19	No	No	No	Banana, tomato	No
H20	Epirus	Multiple sclerosis, dyslipidemia	Peginterferon beta-1A	Covid-19	Ezetimibe, fluoxetine, peginterferon beta-1A	Coronary heart disease	No	Bee	Yes
H21	Epirus	No	No	Covid-19	No	No	No	Bee, Sulfur	No
H22	Epirus	No	No	Covid-19	No	No	No	No	No
H23	Epirus	No	No	Covid-19	No	No	No	No	No

Human I.D.	Region	Current health disorders / comorbidities	Immunosuppression	Vaccination	Concomitant therapies	Family history	Surgical procedures	Allergies	Previous hospitalization
H24	Epirus	No	No	Covid-19	No	No	No	No	No
H25	Epirus	No	No	Covid-19	No	No	No	No	No

Table S7. Antibiotic use in kitchen staff.

Human I.D.	Region	Consumed antibiotics	Reasons for antibiotic consumption	Stock of antibiotics in house	Use of antibiotics before their expiration	Frequency and easiness of antibiotic consumption	Antibiotic consumption after doctor's prescription	Microbial culture test and antibiogram performed	Guidance by doctor
H1	Central Macedonia	No	Treatment	Yes (room temperature, refrigerator)	Yes	Never (only when absolutely necessary)	Always	Never	Partial
H2	Central Macedonia	Moxifloxacin (Winter, Spring), Amoxicillin / clavulanic acid (winter)	Treatment	Yes (room temperature)	Yes	Never (only when absolutely necessary)	Always	Never	Partial
H3	Central Macedonia	Clarithromycin (Winter, Spring)	Treatment	No	Yes	Never (only when absolutely necessary)	Always	Never	Partial
H4	Central Macedonia	Cefuroxime (Winter)	Treatment	No	Yes	Very often	Usually	Never	Partial
H5	Central Macedonia	No	Treatment	No	Yes	Rare	Always	Never	Partial
H6	Central Macedonia	No	Treatment	Yes (room temperature, refrigerator)	Yes	Never (only when absolutely necessary)	Always	Never	Partial
H7	Central Macedonia	No	Treatment	No	Yes	Never (only when absolutely necessary)	Always	Never	Partial
H8	Central Macedonia	No	Treatment	No	Yes	Never (only when absolutely necessary)	Always	Never	Partial
H9	Central Macedonia	Amoxicillin / clavulanic acid (Autumn)	Treatment	No	Yes	Never (only when absolutely necessary)	Always	Never	Partial

Human I.D.	Region	Consumed antibiotics	Reasons for antibiotic consumption	Stock of antibiotics in house	Use of antibiotics before their expiration	Frequency and easiness of antibiotic consumption	Antibiotic consumption after doctor's prescription	Microbial culture test and antibiogram performed	Guidance by doctor
H10	Central Macedonia	Moxifloxacin (Winter)	Treatment	Yes (room temperature)	Yes	Never (only when absolutely necessary)	Always	Never	Partial
H11	Central Macedonia	No	Treatment	No	Yes	Never (only when absolutely necessary)	Always	Never	Partial
H12	Epirus	No	Treatment	No	Yes	Never (only when absolutely necessary)	Always	Never	Partial
H13	Epirus	Amoxicillin / clavulanic acid (Autumn), Ciprofloxacin (winter)	Treatment	No	Yes	Never (only when absolutely necessary)	Always	Never	Partial
H14	Epirus	No	Treatment	No	Yes	Never (only when absolutely necessary)	Always	Never	Partial
H15	Epirus	Azithromycin (Spring)	Prophylaxis and treatment	No	Yes	Sometimes	Usually	Never	Partial
H16	Epirus	Cefuroxime (Spring)	Prophylaxis and treatment	Yes (room temperature)	Yes	Rare	Always	Never	Partial
H17	Epirus	No	Treatment	Yes (room temperature)	Yes	Never (only when absolutely necessary)	Always	Never	Partial
H18	Epirus	No	Treatment	Yes (room temperature)	Yes	Never (only when absolutely necessary)	Always	Never	Partial
H19	Epirus	Cefuroxime (Summer)	Treatment	Yes (room temperature)	Yes	Never (only when absolutely necessary)	Always	Never	Partial
H20	Epirus	No	Treatment	No	Yes	Never (only when absolutely necessary)	Always	Sometimes	Partial
H21	Epirus	No	Treatment	No	Yes	Never (only when absolutely necessary)	Always	Never	Partial
H22	Epirus	Levofloxacin (Autumn)	Treatment	No	Yes	Never (only when absolutely necessary)	Always	Never	Partial

Human I.D.	Region	Consumed antibiotics	Reasons for antibiotic consumption	Stock of antibiotics in house	Use of antibiotics before their expiration	Frequency and easiness of antibiotic consumption	Antibiotic consumption after doctor's prescription	Microbial culture test and antibiogram performed	Guidance by doctor
H23	Epirus	Ciprofloxacin (Autumn)	Prophylaxis and treatment	No	Yes	Sometimes	Usually	Never	Partial
H24	Epirus	No	Treatment	No	Yes	Never (only when absolutely necessary)	Always	Never	Partial
H25	Epirus	Amoxicillin / clavulanic acid (Winter)	Treatment	No	Yes	Never (only when absolutely necessary)	Always	Never	Partial

Table S8. Description of the collected samples.

Sample			Total number of samples
**Environment**	Surfaces	Chicken cutting boards	8
		Meat cutting boards	6
		Vegetable cutting board	6
		Bread cutting board	2
		Cheese cutting board	3
		Surfaces of food preparation	4
		Surfaces around cooked and ready-to-eat food	7
		Refrigerator surfaces	2
		Surfaces around equipment washing-up	2
	Total		**40**
	Equipment	Bread container	1
		Meat container	5
		Cooked food container	2

		Chicken knife	6
		Meat knife	4
		Vegetable knife	6
		Cheese knife	2
		Food preparation utensils	4
		Sponge towel (chicken)	2
		Sponge towel (washing up)	2
		Sponge towel (food preparation)	3
		Sponge towel (cheese)	1
		Sponges (chicken, food preparation)	2
	Total		**40**
	Sinks	Chicken	8
		Meat	3
		Vegetables	6
		Cheese	4
		Bread	2
		Kitchen staff	5
		Washing up	8
		Food preparation	4
	Total		**40**
	Utensils used by hospital patients	Set of cutlery, dishes and glasses	**40**
	Serving trays		**24**
	Tables		**16**
**Chicken**	Breast		**40**
**Human**	Skin		**40**

Oropharynx	**40**

Table S9. List of questions, regarding medical history and history of antibiotic consumption in humans.

A) General information	B) History of consumption of antibiotics
1. Gender	1. List of antibiotics that were used during the last 3 months (route of administration, for how long and why they were administered)
2. Age	2. What are the usual reasons for the administration of antibiotics (prophylaxis, therapy, metaphylaxis)
3. Occupation	3. Is there a stock of antibiotics in the house? Where are they stored (room temperature, refrigerator)?
4. Place of residence	4. Are antibiotics used within their expiration date?
5. Smoking (how much, how long)	5. How frequent and easily is the consumption of antibiotics?
6. Alcohol consumption (how much, how long)	6. Are antibiotics consumed only after a doctor's prescription?
7. Current health disorders / comorbidities	7. Is a microbial culture test and antibiogram conducted before prescribing antibiotics? If yes, give more information about the isolated strain (which species, resistance profile)
8. Occurrence and type of immunosuppression, time of last treatment	8. Does the doctor provide guidance on the prudent use of antibiotics?
9. Vaccination status	
10. Concomitant therapies (drugs)	
11. Family history	
12. Surgical procedures (type and time of conduction)	

13. Allergies

14. Previous hospitalization

(time and type of hospital unit)

Table S10. Description of clinical strains used in the study.

	**Isolated from:**	**Number of *K. pneumoniae* strains**	**Number of *A. baumannii* strains**
Intensive care unit	Pharyngeal	8	7
	Underarm	2	3
	Bronchial	5	1
	Nasal	1	3
Internal Medicine	Pharyngeal	2	0
	Urine	1	0
Orthopedics	Nasal	0	2
**Total**		**19**	**16**

Table S11. Antibiotic discs used for antibiotic susceptibility with the disc diffusion method.

	E. coli* and *K. pneumoniae		***Acinetobacter* spp.**	
**Antibiotic class**	**Antibiotic**	**Abbreviation & concentration**	**Antibiotic**	**Abbreviation & concentration**
Penicillins	Ampicillin	AM* 10 μg		
	Amoxicillin-clavulanic acid	AMC 20/10 μg		
	Ampicillin-sulbactam	SAM 10/10 μg	Ampicillin-sulbactam	SAM 10/10 μg
	Piperacillin-tazobactam	TPZ 100/10 μg	Piperacillin-tazobactam	TPZ 100/10 μg
	Ticarcillin-clavulanic acid	TIM 75/10 μg	Ticarcillin-clavulanic acid	TIM 75/10 μg
			Piperacillin	PRL 100 μg
Cephalosporins	Cefotaxime	CTX 5 μg	Cefotaxime	CTX 5 μg
	Ceftazidime	CAZ 10 μg	Ceftazidime	CAZ 10 μg
	Cefepime	FEP 30 μg	Cefepime	FEP 30 μg
	Cefoxitin	FOX 30 μg		
Carbapenems	Meropenem	MEM 10μg	Meropenemm	MEM 10μg
	Imipenem	IPM 10 μg	Imipenem	IPM 10 μg
	Ertapenem	ETP 10 μg		

Fluoroquinolones	Ciprofloxacin	CIP 5 μg	Ciprofloxacin	CIP 5 μg
	Levofloxacin	LEV 5 μg	Levofloxacin	LEV 5 μg
Aminoglycosides	Tobramycin	TOB 10μg	Tobramycin	TOB 10μg
	Amikacin	AK 30μg	Amikacin	AK 30μg
	Gentamicin	CN 10 μg	Gentamicin	CN 10 μg
Sulfonamides	Trimethoprim-sulfa-methoxazole	SXT 1.25/23.75μg	Trimethoprim-sulfa-methoxazole	SXT 1.25/23.75μg
Phenicols	Chloramphenicol	CHL 30 μg		
Tetracyclines	Tetracycline	TE 30 μg		
	Doxycycline	DO 30 μg	Doxycycline	DO 30 μg
Macrolides	Azithromycin	AZM 15 μg		

* All antibiotic discs were procured by Oxoid Ltd (United Kingdom)

Table S12. Primers and PCR protocols used for molecular screening of β-lactamase genes.

PCR name	Target	Sequence (5′–3′)	Product (bp)	Primer concentration	Reference
Multiplex I (ESBL)	TEM variants	For: CATTTCCGTGTCGCCCTTATTC	800	0.4 μM	[69]

PCR name	Target	Sequence (5′–3′)	Product (bp)	Primer concentration	Reference
		Rev: CGTTCATCCATAGTTGCCTGAC		0.4 μM	[69]
	SHV variants	For: AGCCGCTTGAGCAAATTAAAC	713	0.4 μM	[69]
		Rev: ATCCCGCAGATAAATCACCAC		0.4 μM	[69]
	OXA-1, OXA-4 and OXA-30	For: GGCACCAGATTCAACTTTCAAG	564	0.4 μM	[69]
		Rev: GACCCCAAGTTTCCTGTAAGTG		0.4 μM	[69]
Multiplex II (ESBL)	CTX-M group 1 variants	For: TTAGGAARTGTGCCGCTGYA	688	0.4 μM	[69]
		Rev: CGATATCGTTGGTGGTRCCAT		0.2 μM	[69]
	CTX-M group 2 variants	For: CGTTAACGGCACGATGAC	404	0.2 μM	[69]
		Rev: CGATATCGTTGGTGGTRCCAT		0.2 μM	[69]
	CTX-M group 9 variants	For: TCAAGCCTGCCGATCTGGT	561	0.4 μM	[69]
		Rev: TGATTCTCGCCGCTGAAG		0.4 μM	[69]

PCR name	Target	Sequence (5′–3′)	Product (bp)	Primer concentration	Reference
Simplex I (ESBL)	CTX-M groups 8 and 25	For: AACRCRCAGACGCTCTAC	326	0.4 µM	[69]
		Rev: TCGAGCCGGAASGTGTYAT		0.4 µM	[69]
Multiplex III (AmpC)	ACC-1 and ACC-2	For: CACCTCCAGCGACTTGTTAC	346	0.2 µM	[69]
		Rev: GTTAGCCAGCATCACGATCC		0.2 µM	[69]
	FOX-1 to FOX-5	For: CTACAGTGCGGGTGGTTT	162	0.5 µM	[69]
		Rev: CTATTTGCGGCCAGGTGA		0.5 µM	[69]
	MOX family (MOX-1, MOX-2, CMY-1, CMY-8 to CMY-11 and CMY-19)	For: GCAACAACGACAATCCATCCT	895	0.2 µM	[69]
		Rev: GGGATAGGCGTAACTCTCCCAA		0.2 µM	[69]
	DHA-1 and DHA-2	For: TGATGGCACAGCAGGATATTC	997	0.5 µM	[69]
		Rev: GCTTTGACTCTTTCGGTATTCG		0.5 µM	[69]

PCR name	Target	Sequence (5′–3′)	Product (bp)	Primer concentration	Reference
					[69]
	CIT family (LAT-1 to LAT-3, BIL-1, CMY-2 to CMY-7, CMY-12 to CMY-18 and CMY-21 to CMY-23)	For: CGAAGAGGCAATGACCAGAC	538	0.2 µM	
		Rev: ACGGACAGGGTTAGGATAGY		0.2 µM	[69]
	EBC family (ACT-1 and MIR-1)	For: CGGTAAAGCCGATGTTGCG	683	0.2 µM	[69]
		Rev: AGCCTAACCCCTGATACA		0.2 µM	[69]
Multiplex IV (ESBL)	GES-1 to GES-9 and GES-11	For: AGTCGGCTAGACCGGAAAG	399	0.3 µM	[69]
		Rev: TTTGTCCGTGCTCAGGAT		0.3 µM	[69]
	PER-1 and PER-3	For: GCTCCGATAATGAAAGCGT	520	0.3 µM	[69]
		Rev: TTCGGCTTGACTCGGCTGA		0.3 µM	[69]
	VEB-1 to VEB-6	For: CATTTCCCGATGCAAAGCGT	648	0.3 µM	[69]

PCR name	Target	Sequence (5′–3′)	Product (bp)	Primer concentration	Reference
		Rev: CGAAGTTTCTTTGGACTCTG		0.3 μM	[69]
Multiplex V (carbapenemase)	IMP variants	For: GGAATAGAGTGGCTTAAYTCTC	232	0.2 μM	[53]
		Rev: GGTTTAAYAAAACAACCACC		0.2 μM	[53]
	VIM variants	For: GATGGTGTTTGGTCGCATA	390	0.2 μM	[53]
		Rev: CGAATGCGCAGCACCAG		0.2 μM	[53]
	SPM variants	For: AAAATCTGGGTACGCAAACG	271	0.2 μM	[53]
		Rev: ACATTATCCGCTGGAACAGG		0.2 μM	[53]
Multiplex VI (carbapenemase)	KPC variants	For: CGTCTAGTTCTGCTGTCTTG	798	0.2 μM	[53]
		Rev: CTTGTCATCCTTGTTAGGCG		0.2 μM	[53]
	NDM variants	For: GGTTTGGCGATCTGGTTTTC	621	0.2 μM	[53]
		Rev: CGGAATGGCTCATCACGATC		0.2 μM	[53]

PCR name	Target	Sequence (5′–3′)	Product (bp)	Primer concentration	Reference
	BIC variants	For: TATGCAGCTCCTTTAAGGGC	570	0.2 μM	[53]
		Rev: TCATTGGCGGTGCCGTACAC		0.2 μM	[53]
	OXA-48 like	For: GCGTGGTTAAGGATGAACAC	438	0.2 μM	[53]
		Rev: FCATCAAGTTCAACCCAACCG		0.2 μM	[53]
Multiplex VII (car-bapenemase)	AIM variants	For: CTGAAGGTGTACGGAAACAC	322	0.2 μM	[53]
		Rev: GTTCGGCCACCTCGAATTG		0.2 μM	[53]
	GIM variants	For: TCGACACACCTTGGTCTGAA	477	0.2 μM	[53]
		Rev: AACTTCCAACTTTGCCATGC		0.2 μM	[53]
	SIM variants	For: TACAAGGGATTCGGCATCG	570	0.2 μM	[53]
		Rev: TAATGGCCTGTTCCCATGTG		0.2 μM	[53]
	DIM variants	For: GCTTGTCTTCGCTTGCTAACG	699	0.2 μM	[53]

PCR name	Target	Sequence (5′–3′)	Product (bp)	Primer concentration	Reference
		Rev: CGTTCGGCTGGATTGATTTG		0.2 µM	[53]
Multiplex VIII (car-bapenemase – *Acineto-bacter* only)	OXA-51-like	For: TAATGCTTTGATCGGCCTTG	353	0.2 µM	[51]
		Rev: TGGATTGCACTTCATCTTGG		0.2 µM	[51]
	OXA-23-like	For: GATCGGATTGGAGAACCAGA	501	0.2 µM	[51]
		Rev: ATTTCTGACCGCATTTCCAT		0.2 µM	[51]
	OXA-24-like	For: GGTTAGTTGGCCCCCTTAAA	246	0.2 µM	[51]
		Rev: AGTTGAGCGAAAAGGGGATT		0.2 µM	[51]
	OXA-58-like	For: AAGTATTGGGGCTTGTGCTG	599	0.2 µM	[51]
		Rev: CCCCTCTGCGCTCTACATAC		0.2 µM	[51]

*For multiplex I – IV and simplex I, the following PCR protocol was performed: initial denaturation at 95°C for 3 minutes, followed by 30 PCR cycles involving denaturation at 95°C for 30 seconds, annealing at 60°C for 40 seconds (54°C for the simplex PCR), and extension at 68°C for 1 minute. A final extension at 68°C for 5 minutes concluded the PCR process [69].

**For multiplex V – VIII, the following PCR protocol was performed: initial denaturation at 95°C for 3 minutes, followed by 30 PCR cycles of denaturation at 95°C for 30 seconds, annealing at 52°C for 40 seconds, and extension at 68°C for 1 minute, with a final extension at 68°C for 5 minutes [51,53].

Table S13. Primers and PCR protocol used for phylogenetic analysis of *E. coli* strains.

PCR name	Target	Sequence (5′-3′)	Product (bp)	Primer concentratio[illegible]
Quadruplex	*chuA*	For: ATGGTACCGGACGAACCAAC	288	0.2 μM
		Rev: TGCCGCCAGTACCAAAGACA		0.2 μM
	yjaA	For: CAAACGTGAAGTGTCAGGAG	211	0.2 μM
		Rev: AATGCGTTCCTCAACCTGTG		0.2 μM
	TspE4.C2	For: CACTATTCGTAAGGTCATCC	152	0.2 μM
		Rev: AGTTTATCGCTGCGGGTCGC		0.2 μM
	arpA	For: AACGCTATTCGCCAGCTTGC	400	0.4 μM
		Rev: TCTCCCCATACCGTACGCTA		0.4 μM
Group E	*arpA*	For: GATTCCATCTTGTCAAAATATGCC	301	0.2 μM
		Rev: GAAAAGAAAAAGAATTCCCAAGAG		0.2 μM
Group C	*trpA*	For: AGTTTTATGCCCAGTGCGAG	219	0.2 μM
		Rev: TCTGCGCCGGTCACGCCC		0.2 μM

* The PCR process began with an initial denaturation at 95°C for 180 seconds, followed by 30 cycles of PCR consisting of denaturation at 95°C for 30 seconds, annealing at 59°C for 20 seconds (for quadruplex PCR and group C) or 57°C for 20 seconds (for group E) and extension at 68°C for 60 seconds, and concluded with a final extension at 68°C for 5 min [70].

Table S14. Quadruplex PCR results and categorization of *E. coli* strains to phylogenetic groups.

arpA	*chuA*	*yjaA*	**TspE4.C2**	**Phylogroup**
+	-	-	-	A
+	-	-	+	B1
-	+	-	-	F
-	+	+	-	B2
-	+	+	+	B2
-	+	-	+	B2
+	-	+	-	A or C*
+	+	-	-	D or E**
+	+	-	+	D or E**
+	+	+	-	E or clade I***
-	-	+	-	Clade I or II
-	476 bp product	-	-	Clade III, IV or V
-	-	-	+	Unknown
-	-	+	+	Unknown
+	-	+	+	Unknown
+	+	+	+	Unknown
-	-	-	-	Unknown

* Screen using C-specific primers. If C+, then Group C, else Group A

** Screen using E-specific primers. If E+, then Group E, else Group D

*** Screen using E-specific primers. If E+, then Group E, else clade I
